# ISUOG Practice Guidelines (updated): role of ultrasound in twin pregnancy

**DOI:** 10.1002/uog.29166

**Published:** 2025-01-15

**Authors:** A. Khalil, A. Sotiriadis, A. Baschat, A. Bhide, E. Gratacós, K. Hecher, L. Lewi, L. J. Salomon, B. Thilaganathan, Y. Ville

**Affiliations:** ^1^ Fetal Medicine Unit St George's Hospital, St George's University of London London UK; ^2^ Second Department of Obstetrics and Gynaecology Aristotle University of Thessaloniki Thessaloniki Greece; ^3^ The Johns Hopkins Center for Fetal Therapy Baltimore MD USA; ^4^ Fetal Medicine Unit St George's Hospital, St George's University of London London UK; ^5^ BCNatal, Hospital Clinic and Hospital Sant Joan de Deu, University of Barcelona, IDIBAPS and CIBERER Barcelona Spain; ^6^ Department of Obstetrics and Fetal Medi‐ cine, University Medical Center Hamburg‐Eppendorf Hamburg Germany; ^7^ Department of Obstetrics and Gynecology, Uni‐ versity Hospitals Leuven Leuven Belgium; ^8^ Hopital Necker‐Enfants Malades, AP‐HP, Université Paris Descartes Paris France; ^9^ Fetal Medicine Unit, St George's Hos‐ pital, St George's University of London London UK; ^10^ Hospital Necker‐Enfants Malades, AP‐HP, Uni‐ versité Paris Descartes Paris France

## Clinical Standards Committee

The International Society of Ultrasound in Obstetrics and Gynecology (ISUOG) is a scientific organization that encourages sound clinical practice, and high‐quality teaching and research, related to diagnostic imaging in women's healthcare. The ISUOG Clinical Standards Committee (CSC) has the remit to develop Practice Guidelines and Consensus Statements as educational recommendations that provide healthcare practitioners with a consensus‐based approach, from experts, for diagnostic imaging. They are intended to reflect what is considered by ISUOG to be the best practice at the time at which they are issued. Although ISUOG has made every effort to ensure that Guidelines are accurate when issued, neither the Society nor any of its employees or members accepts any liability for the consequences of any inaccurate or misleading data, opinions or statements issued by the CSC. The ISUOG CSC documents are not intended to establish a legal standard of care because interpretation of the evidence that underpins the Guidelines may be influenced by individual circumstances, local protocol and available resources. Approved Guidelines can be distributed freely with the permission of ISUOG (info@isuog.org).

## INTRODUCTION

The incidence of multiple pregnancy has increased over the years, mainly due to delayed childbirth and advanced maternal age at conception and the resultant widespread use of assisted reproduction techniques^1^. In addition to often involving the transfer of more than one embryo, *in‐vitro fertilization* increases the frequency of monozygotic twinning^2^. The twin birth rate was reported to have increased in the USA by just under 70% between 1980 (19 per 1000 live births) and 2020 (31 per 1000 live births)^3^, though other reports demonstrated a decline in the twin birth between 2014 and 2018 in both the USA and UK^4^.

Twin pregnancy is associated with a high risk of perinatal mortality and morbidity^5^, ^6^, ^7^, ^8^. There is also an increased risk of maternal complications, such as hypertensive disorders of pregnancy^9^. In 2019, the stillbirth rate was 7.6 per 1000 twin births compared with 3.8 per 1000 singleton births^10^. Preterm birth prior to 37 weeks' gestation occurs in up to 60% of multiple pregnancies, while the risk of very preterm birth prior to 32 weeks is 10 times higher in twin compared with singleton pregnancies (10% *vs* 1%), contributing to the increased risk of neonatal mortality and long‐term morbidity^11^, ^12^, ^13^, ^14^. Compared with singleton pregnancies, twin pregnancies are at increased risk of iatrogenic preterm birth due to the greater incidence of maternal and fetal complications. This risk is significantly higher in monochorionic compared with dichorionic pregnancy^5^, ^6^, ^7^, ^8^. Yet, multiple pregnancies are often excluded from research studies, with only 8% of trials on fetal growth restriction (FGR), 17% of those on pre‐eclampsia and 2% of those on diabetes including multiple pregnancies^15^. Moreover, the majority of recommendations in national and international guidelines for the management of multiple pregnancy lack high‐quality robust supporting evidence^16^.

Ultrasound assessment of chorionicity, fetal biometry, anatomy, Doppler velocimetry and amniotic fluid volume is used to identify and monitor twin pregnancies at risk of adverse outcomes, such as twin‐to‐twin transfusion syndrome (TTTS) and FGR. As in singletons, impaired fetal growth can be assessed in twins by comparing biometry and Doppler velocimetry parameters against standards for uncomplicated pregnancy.

This guidance will address the role of ultrasound in the care of uncomplicated twin pregnancies and those complicated by TTTS, selective FGR (sFGR), twin anemia–polycythemia sequence (TAPS), twin reversed arterial perfusion (TRAP) sequence, conjoined twins and single intrauterine death (IUD). The document provides guidance on the methods used to determine gestational age and chorionicity, screening for chromosomal and structural abnormalities, and screening for TTTS, TAPS, TRAP sequence, growth abnormalities and the risk of preterm birth. The management of higher‐order multiple pregnancy will be covered in a separate document.

## OUTLINE/SCOPE

- Dating of the pregnancy (determining gestational age)
- Determining chorionicity and amnionicity
- Twin labeling
- Timing, frequency and content of ultrasound assessment
- Screening for aneuploidy
- Prenatal diagnosis of aneuploidy
- Screening for structural abnormalities
- Diagnosis and management of discordant twin pregnancy
- Fetal reduction/selective termination
- Screening for the risk of preterm birth
- Screening, diagnosis and management of FGR
- Management of twin pregnancy complicated by single IUD
- Complications unique to monochorionic twin pregnancy
  – Screening, diagnosis, staging and management of TTTS
  – Screening, diagnosis and management of TAPS
  – Management of TRAP sequence
  – Management of monochorionic monoamniotic (MCMA) twin pregnancy
  – Diagnosis and management of conjoined twins

## IDENTIFICATION AND ASSESSMENT OF EVIDENCE

The Cochrane Library and Cochrane Register of Controlled Trials were searched for relevant randomized controlled trials (RCTs), systematic reviews and meta‐analyses, and a search of MEDLINE from 1966 to 2022 was carried out. The date of the last search was 31 December 2022. In addition, relevant conference proceedings and abstracts were searched. Databases were searched using the relevant MeSH terms, including all subheadings. This was combined with a keyword search using ‘twin’, ‘multiple’, ‘pregnancy’, ‘ultrasound’, ‘twin‐to‐twin transfusion syndrome’, ‘fetal growth restriction’, ‘twin anemia polycythemia sequence’, ‘twin reversed arterial perfusion’, ‘acardiac twin’, ‘monochorionic monoamniotic’, ‘conjoined’ and ‘demise’. The National Library for Health and the National Guidelines Clearing House were also searched for relevant guidelines and reviews. Gray (unpublished) literature was identified through searching the websites of health technology assessment and health technology assessment‐related agencies, clinical practice guideline collections and clinical trial registries. The search was limited to the English language. When possible, recommendations are based on, and explicitly linked to, the evidence that supports them, while areas lacking evidence are annotated as ‘good practice points’. Details of the grades of recommendation and levels of evidence used in these Guidelines are given in Appendix 1.

## RECOMMENDATIONS

### Dating of twin pregnancy

- Twin pregnancies conceived spontaneously should ideally be dated prior to 13 + 6 weeks of gestation (**GRADE OF RECOMMENDATION: D**).
- In twin pregnancies conceived spontaneously, the larger of the two crown–rump lengths (CRLs) should be used to estimate gestational age (**GRADE OF RECOMMENDATION: C**).
- Fetal head circumference of the larger twin should be used to date the pregnancy at or beyond 14 weeks' gestation (**GRADE OF RECOMMENDATION: D**).
- Twin pregnancies conceived via *in‐vitro* fertilization should be dated using the age of the embryo and the date of transfer (**GRADE OF RECOMMENDATION: C**).

The most common practice for dating twin pregnancies is to use the CRL of the larger twin in the first trimester. Some studies have recommended the use of the smaller CRL or the mean CRL, which takes into account both fetuses^17^, ^18^, ^19^, ^20^, as studies of pregnancies conceived via assisted reproductive technology have shown that the CRL of the smaller twin correlates best with the known gestational age. The disadvantage of using the smaller CRL is the potential for the operator to believe that, in CRL‐discordant pairs, the larger twin is large‐for‐gestational age, therefore being falsely reassured that the smaller twin is growing appropriately. One study showed that using the larger CRL did not increase the proportion of neonates classified as small‐for‐gestational age (SGA)^20^. Recommending the use of the smaller CRL would entail a significant change in practice. Generally, it would alter the due date by only a few days, and it is uncertain whether this would result in any improvement in clinical outcomes. Therefore, pending further evidence to inform this question, the recommendation is to continue with the current practice of using the CRL of the larger twin to date twin pregnancies in the first trimester.

If the woman presents after 14 weeks' gestation, the head circumference of the larger twin should be used to date the pregnancy.

### Determining chorionicity and amnionicity in twin pregnancy

- Chorionicity should be determined prior to 13 + 6 weeks of gestation based on as many ultrasound characteristics as possible, including the entire intertwin septum (at the site of insertion of the amniotic membrane into the placenta, using the lambda sign or the T sign), membrane thickness and number of placental masses (**GRADE OF RECOMMENDATION: D**).
- An ultrasound image demonstrating the chorionicity should be kept in the medical records for future reference (**GOOD PRACTICE POINT**).
- If it is not possible to determine chorionicity by transabdominal or transvaginal ultrasound in the routine setting, a second opinion should be sought from a tertiary referral center (**GOOD PRACTICE POINT**).
- In monochorionic twin pregnancies, amnionicity can be determined from 8 weeks onwards, when the amniotic sac becomes visible on ultrasound scan. MCMA twin pregnancies should be referred to a tertiary center with expertise in their management (**GOOD PRACTICE POINT**).

**Figure 1:**
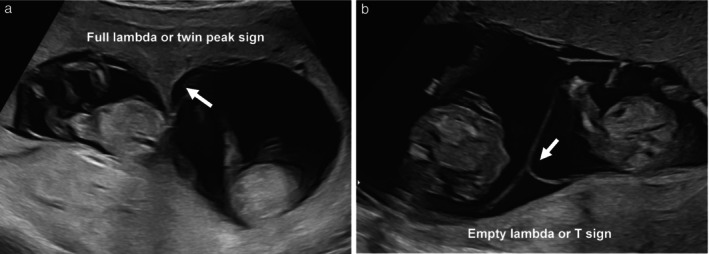
Ultrasound images in the first trimester of: (a) a dichorionic diamniotic twin pregnancy, in which the twins are separated by a thick layer of fused chorionic membranes; and (b) a monochorionic diamniotic twin pregnancy, in which the twins are separated by only two thin amniotic layers. In monochorionic twins, the base of the insertion may still be triangular (empty lambda/T sign (arrow)); however, it does not contain chorion and should not be confused with the full lambda/twin peak sign (arrow) of dichorionic twins.

Every effort should be made to determine the chorionicity of a twin pregnancy. Chorionicity should be determined before 13 + 6 weeks of gestation using the ultrasound features of the intertwin septum (Figure 1). It is important to examine the entire intertwin septum carefully. In dichorionic diamniotic (DCDA) twin pregnancy, the twins are separated by a thick layer of fused chorionic membranes, with two thin amniotic layers, one on each side, giving the appearance of a ‘full lambda’ or ‘twin peak sign’, compared with only two thin amniotic layers separating the two fetuses in monochorionic diamniotic (MCDA) twin pregnancy (T‐sign or empty lambda sign). In women presenting for the first time after 14 weeks of gestation, chorionicity is best determined using the same ultrasound signs, in particular by counting the membrane layers, and noting whether the fetal sex is discordant. The reliability of the number of placental masses is questionable, as dichorionic placentae are commonly adjacent to each other, appearing as a single mass, and 3% of monochorionic twin pregnancies have two placental masses on ultrasound, the presence of which does not preclude the presence of vascular anastomoses^21^. Conversely, approximately 5% of apparently monochorionic twins were reported to be dizygotic in a Danish series^22^, and this phenomenon is more common in conceptions after assisted reproduction^23^. It is likely that using a combination of ultrasound features, rather than a single feature, would be more accurate^1^.

If it is not possible to determine chorionicity by transabdominal ultrasound imaging, this should be attempted using transvaginal sonography. If it is still not possible to determine chorionicity, a second opinion should be sought from a tertiary referral center. If the center is uncertain about the chorionicity, it is safest to classify the pregnancy as monochorionic^1^ (**EVIDENCE LEVEL: 3**).

In monochorionic twin pregnancies, amnionicity (i.e. whether or not the twins share the same amniotic sac) can be determined from 8 weeks onwards, when the amniotic sac becomes visible on ultrasound scan. In case of doubt, absence of the intertwin membrane is best confirmed by transvaginal scan. Another useful finding is demonstration of cord entanglement, which is almost universal in MCMA twin pregnancy, using color and pulsed‐wave Doppler ultrasound. Using pulsed‐wave Doppler, two distinct arterial waveform patterns with different heart rates are seen within the same sampling gate (**EVIDENCE LEVEL: 4**). Pseudo‐ or partial monoamnionicity is a term used to describe MCDA twin pregnancy in which the intertwin membrane has ruptured spontaneously. The term iatrogenic monoamnionicity is used when the intertwin septum in MCDA twin pregnancy is disrupted as a complication of amniocentesis or other invasive fetal procedure^24^, ^25^.

All MCMA twin pregnancies should be referred to a tertiary center with expertise in their management^1^. It is recommended that an ultrasound image of the intertwin septum demonstrating the chorionicity is stored electronically and that a hard copy is added to the medical records. As determination of chorionicity and amnionicity is most accurate in the first trimester, when the amnion and chorion have not yet fused, the first‐trimester scan is paramount in twin pregnancy (**EVIDENCE LEVEL: 4**).

### Labeling of twin fetuses

- The labeling of twin fetuses should follow a reliable and consistent strategy and should be documented clearly in the woman's notes (**GOOD PRACTICE POINT**).
- The labeling of twin fetuses should be based on the lateral or vertical orientation of the gestational sacs and include as many parameters as possible (**GOOD PRACTICE POINT**).

It is important to follow a reliable, consistent strategy for antenatal twin labeling. Options include: labeling according to their site, either right and left, or lower and upper; or mapping in the first trimester according to the insertion of their cords relative to the placental edges and membrane insertion. In some healthcare settings, Twin A is the fetus on the right side, while Twin B is the one on the left. Categorical information, i.e. different sex or discordance for structural anomalies, can also be used when present, as they are not likely to change with advancing gestation. This information should be documented clearly in the woman's notes in order to ensure consistent labeling during follow‐up scans^26^. Overall, it is advisable to describe each twin using as many features as possible, so as to enable others to identify them accurately; e.g. ‘Twin A (female) is on the maternal right with a posterior placenta and marginal cord insertion’. For pregnancies with discordance, the labeling should be accompanied by a description such as ‘Twin A, potential recipient’. It is important to acknowledge that labeling is less accurate (or not possible) in MCMA twin pregnancy, particularly in the absence of discordance.

#### The perinatal switch phenomenon

It should be borne in mind that the twins labeled as ‘Twin A’ and ‘Twin B’ during antenatal ultrasound scans may not necessarily be delivered in that order, particularly if the mode of delivery is Cesarean section^27^. It is important to alert parents and healthcare professionals attending the birth to this fact, especially in pregnancies in which the twins are discordant for structural abnormalities that are not obvious on external examination, for example congenital diaphragmatic hernia or cardiac defects. In such cases, an ultrasound scan should be performed just prior to delivery and also before instigating any specific neonatal intervention.

### Routine monitoring of twin pregnancy with ultrasound

- Women with an uncomplicated dichorionic twin pregnancy should have a first‐trimester scan, a second‐trimester anomaly scan and scans every 4 weeks thereafter. Complicated dichorionic twins should be scanned more frequently, depending on the condition and its severity (**GOOD PRACTICE POINT**).
- Uncomplicated monochorionic twins should have a first‐trimester scan and be scanned every 2 weeks after 16 weeks, in order to detect TTTS in a timely manner. Complicated monochorionic twins should be scanned more frequently, depending on the condition and its severity (**GRADE OF RECOMMENDATION: C**).

**Figure 2:**
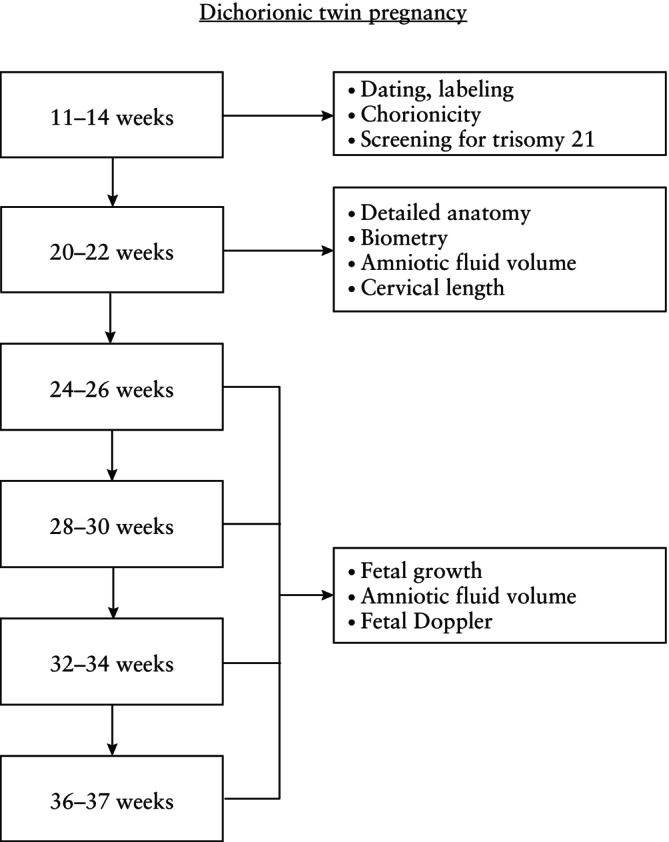
Ultrasound monitoring pathway in uncomplicated dichorionic twin pregnancy.

**Figure 3:**
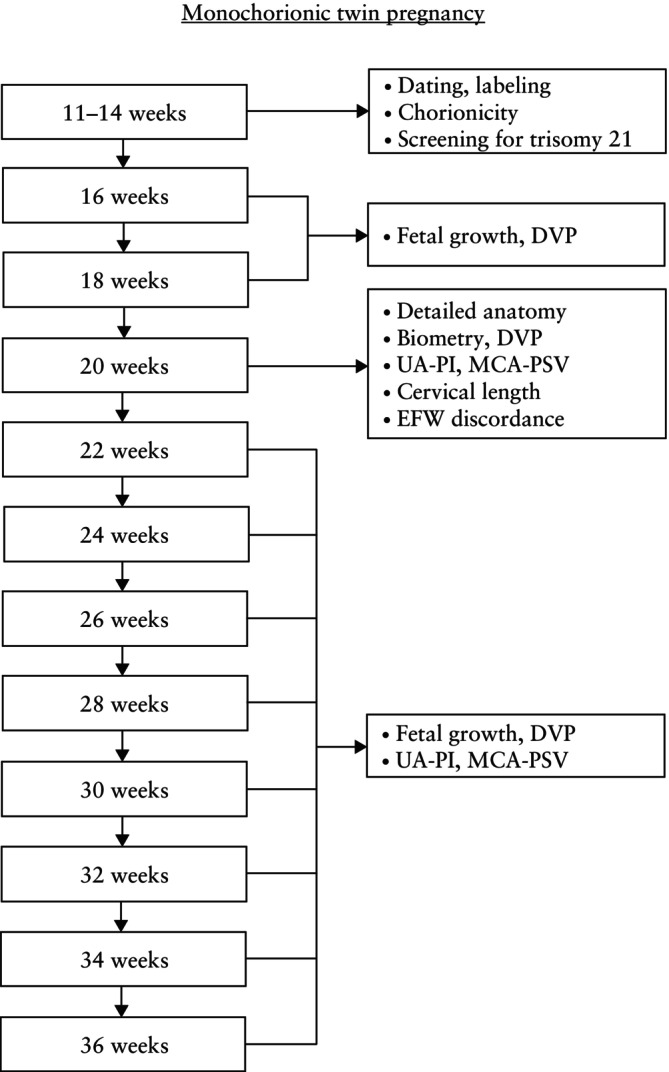
Ultrasound monitoring pathway in uncomplicated monochorionic twin pregnancy. DVP, deepest vertical pocket; EFW, estimated fetal weight; MCA, middle cerebral artery; PI, pulsatility index; PSV, peak systolic velocity; UA, umbilical artery.

In an uncomplicated dichorionic twin pregnancy, ultrasound imaging should be performed in the first trimester, again at around 20 weeks' gestation (second‐trimester anomaly scan) and every 4 weeks thereafter, unless a complication is detected which might require more frequent scans (Figure 2)^1^. In an uncomplicated monochorionic twin pregnancy, an ultrasound scan should be performed in the first trimester, followed by scans every 2 weeks from 16 weeks onwards, as timely detection of TTTS has been shown to improve perinatal outcome (Figure 3)^28^, ^29^ (**EVIDENCE LEVEL: 4**).

Currently, the optimal gestational age for delivery of uncomplicated dichorionic twins is considered to be between 37 + 0 and 37 + 6 weeks, and that for uncomplicated monochorionic twins between 36 + 0 and 36 + 6 weeks, as prolongation of pregnancy beyond this stage may increase the risk of perinatal mortality^30^.

At each ultrasound assessment, the following should be evaluated: fetal biometry, amniotic fluid volume and umbilical artery (UA) Doppler (the latter from 20 weeks' gestation in monochorionic and from 24 weeks' gestation in dichorionic twin pregnancies) for both twins. Discordance in estimated fetal weight (EFW) should be calculated and documented at each scan from 20 weeks onwards. In monochorionic twin pregnancy, middle cerebral artery (MCA) peak systolic velocity (PSV) should be recorded from 20 weeks onwards, in order to screen for TAPS. In MCDA twins, the amniotic fluid volume (deepest vertical pocket (DVP)) should be assessed and documented at each ultrasound scan, to screen for TTTS.

### Screening for chromosomal abnormalities in twin pregnancy

- Screening for trisomy 21 in twin pregnancy should be offered in the first trimester. The most accurate test that can be offered from 10 weeks' gestation uses cell‐free DNA (cfDNA) in the maternal blood. The detection rate (DR) of non‐invasive prenatal testing (NIPT) for trisomy 21 using cfDNA may be slightly lower in twins than in singletons, but it is the most accurate method of screening for trisomy 21 in twin pregnancy. Data on the screening performance using NIPT for other trisomies (trisomies 18 and 13) are limited and further research is needed (**GRADE OF RECOMMENDATION: B**).
- If NIPT is not available, screening for trisomy 21 should be performed in the first trimester using the combined test (nuchal translucency thickness (NT), free beta‐human chorionic gonadotropin (β‐hCG) level and pregnancy‐associated plasma protein‐A (PAPP‐A) level). An alternative is a combination of maternal age and NT, depending on the clinical context and/or health setting (**GRADE OF RECOMMENDATION: B**).
- In case of a vanished twin, just NT in combination with maternal age should be used for risk estimation. An alternative could be NT in combination with maternal age and free β‐hCG level (**GRADE OF RECOMMENDATION: B**).

In twin pregnancy, screening for trisomy 21 can be performed in the first trimester using the combined test, which includes maternal age, NT measurement and serum free β‐hCG and PAPP‐A levels^1^. An alternative is the combination of maternal age and the NT recorded between 11 + 0 and 13 + 6 weeks of gestation, depending on the clinical context and/or healthcare setting.

The phenomenon of a vanishing twin occurs in around one in five of all twin pregnancies and is more common in those conceived via assisted reproductive technology^31^, ^32^. In a retrospective study comparing maternal serum free β‐hCG and PAPP‐A levels at 11–13 weeks' gestation in dichorionic pregnancies with a vanishing twin (an empty gestational sac or a dead embryo) with those in normal singleton pregnancies matched for method of conception and gestational age at examination, the levels of maternal serum free β‐hCG were similar, while the PAPP‐A levels were higher^33^. Using a modeling approach, similar performance of screening for trisomy 21 could be achieved in pregnancies with, compared to those without, a vanishing twin, provided that appropriate adjustments were made to the level of PAPP‐A to account for the interval between embryonic demise and blood sampling. The researchers proposed that screening in twin pregnancies with a vanishing twin could potentially rely on a combination of maternal age, NT measurement and serum free β‐hCG, as in singleton pregnancy, without the use of serum PAPP‐A, and that maternal serum PAPP‐A level could be included only after appropriate adjustment for the interval between embryonic demise and blood sampling^33^. Prospective validation of this approach is needed before its routine implementation in clinical practice.

The risk of trisomy 21 in monochorionic and thus monozygotic twin pregnancy is calculated per *pregnancy* based on the average risk of both fetuses, whereas in dichorionic twin pregnancy the risk is calculated per *fetus*, because around 90% are dizygotic. It has been assumed previously that monochorionic twins would have the same chance of having Down syndrome as singletons, and dichorionic twins would have double the risk of at least one twin being affected^34^. However, this does not appear to be the case. It has been found that the observed‐to‐expected ratio of Down syndrome in twins is lower than that in singletons: 33.6% for monozygotic, 75.2% for dizygotic and 70.0% for all twins^35^, ^36^ (**EVIDENCE LEVEL: 2++**).

The DR of the combined first‐trimester test for Down syndrome may be lower in twin compared with singleton pregnancy^1^. However, a meta‐analysis reported similar performance (89% for singletons, 86% for dichorionic twins and 87% for monochorionic twins, at a false‐positive rate (FPR) of 5%)^37^ (**EVIDENCE LEVEL: 2++**).

The likelihood of being offered invasive testing on the basis of a combined screening result is greater in twin compared with singleton pregnancy^1^. Moreover, invasive testing may carry a greater risk in twins^38^, ^39^, ^40^. A meta‐analysis showed that the overall procedure‐related loss rate following chorionic villus sampling (CVS) in twin pregnancy was 3.8%, and following amniocentesis it was 3.1%^38^. Other reports have cited lower loss rates: 2% following CVS and 1.5–2% following amniocentesis^41^. The risk was found to be similar for transabdominal *vs* transcervical approaches, use of a single‐needle *vs* double‐needle system, and single *vs* double uterine entry^23^, and may be attributable more to background risk factors rather than to the procedure itself^42^, ^43^ (See also ‘Invasive prenatal diagnosis in twin pregnancy’ section, below.) (**EVIDENCE LEVEL: 2++**).

Screening and diagnostic testing for trisomies is more complex in twin compared with singleton pregnancy. It is important, therefore, that counseling prior to testing is provided by healthcare professionals with expertise in this area^1^. It is important to inform in advance women and their partners regarding the potentially complex decisions that they will need to make on the basis of the results of combined screening, bearing in mind the increased risk of invasive testing in twins, the possible discordance between dichorionic twins for fetal aneuploidy, and the risks of selective fetal reduction^1^.

NIPT of fetal cfDNA in maternal blood for risk assessment for fetal trisomy 21 is now commonly used in clinical practice. It has the potential to overcome many of these complex issues, because it has a much higher DR and lower FPR than does the combined test^44^. In singletons, NIPT has a DR of > 99% for trisomy 21, with a FPR of 0.04%^45^. Several factors can affect the use of NIPT in twin pregnancy. First, in dichorionic twins, aneuploidy is usually discordant; if the normal twin contributes a greater fetal fraction to the cfDNA in the maternal blood, this can lead to a false‐negative result^46^, ^47^. Second, NIPT has a higher failure rate in twin pregnancy, with dichorionicity, conception by *in‐vitro* fertilization and greater maternal weight having been identified as significant predictors of failure of NIPT^46^, ^48^. Third, single‐twin demise can render unreliable the results of NIPT. These early deaths are more likely to occur in an aneuploid fetus, and this can lead to unreliable results due to the continued release of cfDNA from the demised twin into the maternal circulation^49^, ^50^.

Several studies have investigated the performance of NIPT in twin pregnancy. For trisomy 21, the reported DR ranges from 94% to 100%, with a failure rate of 2.9% to 9.4%^45^, ^46^, ^47^, ^51^. For trisomies 18 and 13, the DR was 60% in twins^47^, compared with 97.9% and 99%, respectively, in singletons^45^. A recent study that recruited over 1000 twin pregnancies concluded that NIPT using cfDNA testing is the most accurate screening test for trisomy 21 in twin pregnancy, with a DR of 100% and a FPR of 0%, and a low failure rate of 0.3% (lower than that reported in other studies)^52^. However, the performance of this test for trisomies 18 and 13 was less accurate^52^. An updated meta‐analysis on this topic included 137 twin pregnancies with trisomy 21, 50 with trisomy 18 and 11 with trisomy 13, and over 7500 twin pregnancies unaffected by these three trisomies^53^. The pooled weighted DR and FPR for trisomy 21 were 99.0% and 0.02%, respectively; the equivalent figures for trisomy 18 were 93% and 0.01%, respectively, and those for trisomy 13 were 95% and 0.10%, respectively. In summary, NIPT using cfDNA is the most accurate screening test for trisomies in twin pregnancy. Nevertheless, the number of reported cases of a trisomy in twin pregnancy diagnosed using cfDNA testing remains low, and further evidence is needed (**EVIDENCE LEVEL: 2++**).

### Invasive prenatal diagnosis in twin pregnancy

- CVS should be preferred to amniocentesis in dichorionic twin pregnancy, as it provides an earlier prenatal diagnosis (**GRADE OF RECOMMENDATION: D**).
- In MCDA twin pregnancy complicated by discordant anomaly, the option of dual amniocentesis should be considered (**GRADE OF RECOMMENDATION: D**).

When invasive testing for chromosomal or genetic analysis of twins is indicated or desired, it should be carried out by a fetal medicine expert. CVS is preferred in dichorionic twin pregnancy because it can be performed earlier than amniocentesis. Earlier diagnosis of any aneuploidy is particularly important in dichorionic twin pregnancy, given the lower risk of selective termination in the first compared with the second trimester^54^, ^55^.

It is important to map carefully the position of the twins within the uterus. During amniocentesis in monochorionic twins, if monochorionicity has been confirmed before 14 weeks' gestation and the fetuses appear concordant for growth and anatomy, it is acceptable to sample only one amniotic sac. Otherwise, both amniotic sacs should be sampled because of the possibility of rare discordant chromosomal anomalies in monochorionic pregnancy. CVS in monochorionic pregnancy will sample only the single placenta, so will miss these rare discordant chromosomal anomalies. Discordance for most of the common human aneuploidies (trisomies 13, 18 and 21, Turner syndrome and triploidy) has been reported in monochorionic twin pairs^56^. In the event of heterokaryotypic monochorionic pregnancy, selective reduction by umbilical cord occlusion can be offered from 16 weeks onwards, with a survival rate of more than 80% for the healthy twin^57^, ^58^. When monochorionic twins are discordant for an abnormality, prior to invasive testing a discussion should take place regarding the complexity of selective termination, should this become necessary^58^ (**EVIDENCE LEVEL: 3**).

A 2012 meta‐analysis^38^ of amniocentesis in twin pregnancies reported a pooled 3.07% pregnancy loss rate, and a 2.54% loss rate before 24 weeks; for case–control studies, the pooled loss rates for twin pregnancies undergoing amniocentesis and for control twins were 2.59% *vs* 1.53% (relative risk, 1.81 (95% CI, 1.02–3.19)). No difference was found between single *vs* double uterine entry (**EVIDENCE LEVEL: 2+**). The same meta‐analysis^38^, albeit with limited data for CVS, reported a pooled loss rate of 3.84% after CVS in twins. There were no significant differences between the transabdominal and transcervical approach, use of a single‐needle system *vs* a double‐needle system, or single uterine entry *vs* double uterine entry (**EVIDENCE LEVEL: 2+**). No significant differences in loss rates have been reported between CVS and amniocentesis in retrospective studies comparing the two methods. A study including twin pregnancy data from the years 1984–1990 reported a 3.2% loss rate after CVS *vs* 2.9% after amniocentesis^59^ (**EVIDENCE LEVEL 2+**). A more recent study found a non‐significant difference, reporting loss rates of 3.85% and 4.0% after CVS and amniocentesis, respectively^60^ (**EVIDENCE LEVEL: 2+**). There are insufficient data to compare the loss rate related to CVS with the background risk in twins.

A meta‐analysis published in 2020^61^ compared directly outcomes between women with twin pregnancy undergoing amniocentesis and those not undergoing amniocentesis, and between women undergoing CVS and those not undergoing CVS. It was found that, compared to the background rate of fetal loss, in pregnancies undergoing amniocentesis, there was no significant difference in the rate of fetal loss before 24 weeks of gestation (odds ratio (OR), 1.59; *P* = 0.06) or within 4 weeks after the procedure (OR, 1.38, *P* = 0.3). Overall, the pooled rate of fetal loss was 2.4% (95% CI, 1.4–3.6%) in twin pregnancies undergoing amniocentesis compared with 2.4% (95% CI, 0.9–4.6%) in those not undergoing amniocentesis. Similarly, there was no significant difference compared with the background rate in either overall fetal loss (OR, 1.61; *P* = 0.5) or fetal loss before 24 weeks of gestation (OR, 1.61; *P* = 0.5) following CVS. Overall, the pooled rate of fetal loss was 2.0% (95% CI, 0.0–6.5%) in twin pregnancies undergoing CVS compared with 1.8% (95% CI, 0.3–4.2%) in those not undergoing CVS.

Those undergoing invasive testing may represent a selected population already at increased risk of miscarriage; two recent multicenter studies attempted to control for this while assessing the CVS procedure‐related risk of miscarriage in twin pregnancy^42^, ^43^. The first study^42^ used multivariable logistic regression analysis with backward stepwise elimination, adjusting for maternal and pregnancy characteristics, including maternal age, racial origin and weight, method of conception, smoking status, parity, chorionicity, intertwin discordance in CRL, fetal NT ≥ 95^th^ percentile and free β‐hCG and PAPP‐A multiples of the median (MoM). The authors reported that, after adjustment for maternal and pregnancy characteristics, CVS did not contribute significantly to the risk of fetal loss. They also found no significant association between fetal loss and the number of intrauterine needle insertions or needle size (**LEVEL OF EVIDENCE 2++**). The second of these studies^43^, from the same group, assessed the risk of death of at least one fetus in twin pregnancies that had CVS and those that did not, after propensity score matching (1:1 ratio) which created two comparable groups by balancing the maternal and pregnancy characteristics that led to CVS being performed. The authors reported that there was at least one fetal loss in 29 (11.2%) cases in the CVS group and in 35 (13.6%) cases in the matched non‐CVS group (OR, 0.81; 95% CI, 0.48–1.35; *P* = 0.415). However, there was a significant interaction between the risk of fetal loss after CVS and the background risk of fetal loss: when the background risk was higher, the risk of fetal loss after CVS was lower (OR, 0.46; 95% CI, 0.23–0.90), while, in pregnancies with a lower background risk of fetal loss, the risk of fetal loss after CVS was higher (OR 2.45; 95% CI, 0.95–7.13) (**LEVEL OF EVIDENCE 2++**).

In summary, the current evidence suggests that the contribution of amniocentesis or CVS to the risk of fetal loss in twin pregnancy is likely to be small, with procedure‐related loss rates of less than 1% (though, paradoxically, the risk might be a little greater in pregnancies at lower background risk of fetal loss).

The technique for amniocentesis and CVS in twin pregnancies is described in more detail in the ISUOG Practice Guidelines for invasive procedures for prenatal diagnosis^62^. In a dichorionic twin pregnancy, sampling of both amniotic sacs is recommended. There is a small (1.8%) risk of sampling the same sac twice with the two‐puncture technique (one per sac). Using the single‐puncture technique with intertwin membrane passage, the first 1–2 mL of amniotic fluid sampled after intertwin membrane passage should be discarded to avoid contamination from the first twin. If sampling of two sacs is clinically indicated, as in the case of monochorionic twin pregnancy, the two‐puncture technique is recommended to avoid iatrogenic monoamnionicity (**EVIDENCE LEVEL: 4**). When performing CVS, it is recommended to sample the placenta near the cord insertion and to avoid the area around the dividing membrane in order to avoid unreliable or inaccurate results (which have been reported in 3–4% of cases) (**EVIDENCE LEVEL: 4**). A single‐sampling approach around the amniotic equator is a reasonable option in monochorionic twin pregnancy (**EVIDENCE LEVEL: 4**). Determination of zygosity should be recommended for the laboratory analysis. It is preferred that the same operator performs the invasive diagnosis and the selective termination procedure, if needed, taking into account local protocols and the resources available.

### Implications of discordance in NT or CRL in the first trimester in twin pregnancy

- The management of twin pregnancy with CRL discordance ≥ 10% or NT discordance ≥ 20% should be discussed with a fetal medicine expert in accordance with local guidelines and resource availability (**GOOD PRACTICE POINT**).

Although some studies have reported an association between first‐trimester intertwin discordance in NT or CRL, or reversed a‐wave in the ductus venosus (DV), and the development of TTTS, their predictive value is poor^26^, ^63^, ^64^, ^65^, ^66^. NT discordance of 20% had a sensitivity of 52–64%, specificity of 78–80%, positive predictive value of 50% and negative predictive value of 86% for the development of TTTS^67^, ^68^. Discordance in NT of ≥ 20% is found in around 25% of monochorionic twin pregnancies, and the risk of early IUD or development of severe TTTS in these cases is more than 30%^68^. The risk of complications is less than 10% if the NT discordance is <20%^68^. An abnormal DV (reversed a‐wave in at least one of the fetuses) will pick up only 38% of all monochorionic twin pregnancies that will subsequently develop TTTS, and, of those predicted to be at high risk, only 30% will ultimately develop TTTS^65^. Similarly, although intertwin discordance in CRL at 11–13 weeks' gestation is significantly associated with the risk of pregnancy loss ≥ 24 weeks, birth‐weight discordance and preterm birth prior to 34 weeks' gestation, again, the predictive value is poor^69^, ^70^. Nevertheless, the management of twin pregnancy with CRL discordance ≥ 10% or NT discordance ≥ 20% should be discussed with a fetal medicine expert in accordance with local guidelines and depending on resource availability, and in these pregnancies there should be detailed ultrasound assessment and possibly testing for aneuploidy if fetal abnormalities are identified. The risk of fetal abnormality was found to be 25% in dichorionic twin pregnancies with CRL discordance ≥ 10%, compared with 4% in pregnancies with CRL discordance < 10%^71^. Also, CRL discordance at 7 + 0 to 9 + 6 weeks' gestation is a predictor of the risk of single fetal demise in the first trimester (DR, 74% for a FPR of 5%)^72^ (**EVIDENCE LEVEL: 2++**).

### Ultrasound screening for structural abnormalities in twin pregnancy

- Twin fetuses should be assessed for the presence of any major anomalies at the first‐trimester scan, and a routine second‐trimester (anomaly) scan should be performed at around 20 (18–22) weeks' gestation (**GOOD PRACTICE POINT**).
- Fetal cardiac assessment should be performed in monochorionic twins. The operator performing this assessment will depend on the resources and healthcare setting (**GOOD PRACTICE POINT**).

At the first‐trimester scan (between 11 + 0 and 13 + 6 weeks' gestation), twin fetuses should be assessed for the presence of any major anomalies^73^. Routine second‐trimester ultrasound screening for anomalies in twins should be performed by an experienced operator at around 20 (18–22) weeks' gestation^1^, ^74^. This scan may be more difficult than usual because of the presence of a second fetus, and it is important to allow adequate time (minimum, 45 min^16^). The risk of fetal anomaly is greater in twin compared with singleton pregnancy^75^. The anomaly rate per fetus in dizygotic twins is about 30% higher than that in singletons (3.2% *vs* 2.4%)^76^, whereas it is two‐to‐three times higher in monozygotic twins. In around 1 in 30 dichorionic, 1 in 15 MCDA and 1 in 4 monoamniotic twin pregnancies, there is a major congenital anomaly that typically affects only one twin^77^, ^78^, ^79^ Therefore, detailed screening for anomalies should be performed in monochorionic twin pregnancy, bearing in mind that brain and cardiac abnormalities might become more obvious in the third trimester of pregnancy. Abnormalities associated with twins include neural tube defects, anterior abdominal wall defects, facial clefts, brain abnormalities, cardiac defects and gastrointestinal anomalies. Cardiac anomalies are more common in monochorionic twins than in singletons and than in dichorionic twins^80^, ^81^. Therefore, fetal cardiac assessment should be performed according to ISUOG guidelines^82^, including assessment of laterality, situs and four‐chamber, ventricular outflow tract and aortic arch views. It is important to make the woman aware of the limitations of ultrasound screening, which vary according to the type of anomaly. The benefits of screening for fetal anomalies in the second trimester include giving parents the chance to prepare for the birth of a baby with a potential problem, offering them the option of termination, allowing transfer to a specialist center for delivery and, potentially, facilitating intrauterine therapy^1^ (**EVIDENCE LEVEL: 3**).

### Managing twin pregnancy discordant for fetal anomaly

- Twin pregnancies discordant for fetal anomaly should be referred to a regional fetal medicine center (**GOOD PRACTICE POINT**).

Approximately 4% of twin pregnancies (3.4% of DCDA and 6% of monochorionic twin pairs) have an anomaly affecting only one fetus, leading to the challenging decision between expectant management and selective termination of the affected twin. Even in monozygotic twins, concordance for a structural anomaly is found in fewer than 20% of cases. Such pregnancies should be referred to a regional fetal medicine center for further management^79^. In monochorionic twins discordant for a structural abnormality, discordant aneuploidy is very rare (though not impossible). In these situations, expert ultrasound assessment in a tertiary center, with invasive fetal chromosomal or genetic testing if indicated, and a discussion of the likely prognosis for both the affected and the normal twin, are essential. For conditions that are lethal and carry a high risk of intrauterine demise, conservative management is preferred in dichorionic twins, whereas in monochorionic twin pregnancy this would warrant intervention to protect the healthy cotwin against the adverse effects of spontaneous demise of the other. However, a recent cohort study evaluated the outcome of the healthy cotwins in groups of discordant monochorionic twins undergoing expectant management *vs* selective feticide by fetoscopy or bipolar cord coagulation^83^, and found no significant difference in the live‐birth rate between the two management groups (88.5% *vs* 82.7%; *P* = 0.87). Therefore, the optimal management strategy for monochorionic twins discordant for anomalies remains controversial.

### Selective feticide in twin pregnancy

- In dichorionic twin pregnancy, selective feticide is performed by ultrasound‐guided intracardiac or intrafunicular injection of potassium chloride or lignocaine, preferably in the first trimester (**GRADE OF RECOMMENDATION: B**).
- When the diagnosis is made in the second trimester, the woman might opt for late selective termination in the third trimester, if the law permits (**GOOD PRACTICE POINT**).
- Selective feticide in monochorionic twin pregnancy is performed by cord occlusion, intrafetal laser ablation, microwave ablation or radiofrequency ablation (RFA) (**GRADE OF RECOMMENDATION: B**).

The timing of selective termination in twin pregnancy influences the risk of miscarriage and/or preterm birth. This is particularly relevant in twin pregnancies discordant for anomalies, in which selective termination in the second trimester is associated with a higher risk of miscarriage and preterm birth, compared with that in the first trimester^54^. In a recent meta‐analysis, the risk of pregnancy loss prior to 24 weeks was significantly lower in dichorionic twin pregnancies undergoing early (before 18 weeks) compared to late (after 18 weeks) selective termination (1% *vs* 8%)^55^. Similarly, the risk of preterm birth < 32 weeks' gestation was significantly lower in dichorionic twin pregnancies undergoing early compared to late selective termination (3% *vs* 20%)^55^.

When the diagnosis is made in the second trimester, the woman might opt for a late selective termination in the third trimester, if the law permits, when the procedure is associated with a risk of preterm birth rather than fetal loss of the unaffected twin. The pros and cons of each option should be considered (prematurity, fetal loss rate, parental stress, availability of a fetal medicine specialist to perform the procedure in the event of preterm labor, and risk of complications associated with the specific anomaly) (**EVIDENCE LEVEL: 2++**).

Selective feticide in dichorionic twin pregnancy is performed by ultrasound‐guided intracardiac or intrafunicular injection of ‘strong’ potassium chloride or 2% lignocaine. When selective termination of one twin of a monochorionic pair is the choice, injection of potassium chloride is not an option because of the risk to the healthy cotwin. Instead, cord occlusion, intrafetal laser ablation, microwave ablation or RFA of the affected twin is necessary^84^, ^85^, ^86^. This leads to demise of the affected twin while protecting the healthy twin against losing part of its circulating blood volume into the terminated twin following its death. The survival rate of the cotwin following selective termination in monochorionic twin pregnancy is approximately 80%, and the risk of preterm prelabor rupture of the membranes and birth prior to 32 weeks is 20%^85^. The risk of adverse neurological sequelae in the surviving cotwin may also be increased compared with that in uncomplicated pregnancy^85^, ^87^, ^88^, ^89^ (**EVIDENCE LEVEL: 2++**); ISUOG recommends that fetal magnetic resonance imaging (MRI) should be considered in this context^90^.

### Screening for risk of preterm birth in twin pregnancy

- Cervical‐length measurement (ideally transvaginally) is the preferred method of screening for preterm birth in twins; 25 mm is a pragmatic cut‐off between 18 and 24 gestational weeks (**GRADE OF RECOMMENDATION: B**).
- Cervical length should be measured at the anatomy scan and, in case of additional risk factors, once again before 24 weeks (**GRADE OF RECOMMENDATION**: **C**).
- Prophylactic use of progesterone is not recommended for the prevention of preterm birth in unselected twin pregnancy (**GRADE OF RECOMMENDATION: A**).
- Prophylactic use of vaginal progesterone may be considered in twin pregnancy with cervical length ≤ 25 mm (**GRADE OF RECOMMENDATION: C**).
- A combined strategy of physical‐exam‐indicated cerclage, antibiotics and tocolytics may be considered in asymptomatic twin pregnancy with dilated cervix before 24 weeks of gestation (**GRADE OF RECOMMENDATION: C**).
- Cervical cerclage may be considered when cervical length is ≤ 15 mm before 24 weeks of gestation (**GRADE OF RECOMMENDATION: C**).

Both spontaneous and iatrogenic preterm birth are more common in twin than in singleton pregnancy^91^. More than half of twins are born before 37 weeks of gestation (60% and 12% of twin births occur before 37 and 32 weeks of gestation, respectively; these rates are 5.4 and 7.6 times the equivalent rates for singleton pregnancy, respectively)^91^. The rate also depends on chorionicity; the overall rate of birth < 37 weeks for MCMA twin pregnancy is 100%, for MCDA twin pregnancy it is 88.5% and for DCDA twin pregnancy it is 48.6%; the corresponding rates for preterm birth < 32 weeks are 26.8%, 14.2% and 7.4%, respectively^92^.

Cervical length should ideally be measured using transvaginal ultrasound. Asymptomatic women found to have a short cervix at the second‐trimester ultrasound scan are known to be at increased risk of spontaneous preterm birth^93^, ^94^, ^95^. However, the sensitivity of this finding is low, and the cervical‐length cut‐off used to define increased risk of preterm birth is controversial. The cervical‐length distribution in twins is skewed towards shorter lengths. Although the median cervical length is 38 mm, similar to that for singletons, 11% of twin pregnancies have cervical length < 25 mm and 4% have cervical length < 15 mm^96^. A cervical length < 25 mm at 18–24 weeks' gestation in twin pregnancy is a moderate predictor of preterm birth before 34 weeks, but not before 37 weeks^93^, ^94^. In asymptomatic women, a cervical length ≤ 20 mm at 20–24 weeks was the most accurate predictor of preterm birth before 32 and before 34 weeks (pooled sensitivity, 39% and 29%, respectively; pooled specificity, 96% and 97%; positive likelihood ratio, 10.1 and 9.0; and negative likelihood ratio, 0.64 and 0.74). A cervical length ≤ 25 mm at 20–24 weeks had a pooled positive likelihood ratio of 9.6 for the prediction of preterm birth before 28 weeks^93^, ^94^. The predictive accuracy of cervical length for preterm birth was low in symptomatic women^93^, ^94^.

A recent individual patient data (IPD) meta‐analysis highlighted the importance of the timing of cervical‐length screening^95^. If the target is preterm birth < 28 weeks, screening should commence before 18 weeks, regardless of the cervical‐length cut‐off used. For preterm birth between 28 and 32 weeks, the earlier the screening, the lower the cervical‐length cut‐off required to achieve the best prediction. In the common gestational‐age window for cervical‐length screening of 20–22 weeks, the optimal cut‐off to predict preterm birth between 28 and 32 weeks is ∼15 mm, and that for preterm birth between 32 and 36 weeks is ∼35 mm (**EVIDENCE LEVEL: 2++**).

A recent study found that serial measurement of cervical length improved the prediction of preterm birth compared with a single measurement of cervical length made mid‐gestation^97^ (**EVIDENCE LEVEL: 2++**). In women asymptomatic for preterm birth who underwent serial cervical‐length measurements every 2 weeks, starting between 16 and 32 weeks, four patterns of longitudinal change were identified: (1) stable cervix (44%), (2) early and rapid cervical shortening (4%), (3) late cervical shortening (25%) and (4) early cervical shortening with a plateau (27%)^98^. The rates of preterm birth before 34 weeks for these four groups were 11.7%, 44.4%, 20.2% and 14.4%, respectively (**EVIDENCE LEVEL: 2++**). However, such a strategy would clearly place a significant additional burden on clinical resources, and has not been tested in terms of cost‐effectiveness. Therefore, we recommend that cervical length is measured at the anatomy scan and, in case of additional risk factors, once again before 24 weeks.

Current evidence does not suggest that routine screening with fetal fibronectin, insulin‐like growth factor binding protein‐1 (IGFBP‐1) or placental alpha microglobulin‐1 (PAMG‐1) is useful in predicting the risk of preterm birth in twins^93^, ^99^, ^100^, ^101^ (**EVIDENCE LEVEL: 2++**).

Identifying an effective strategy to prevent preterm birth in twin pregnancy has proved challenging. Bed rest, Arabin cervical pessary, cervical cerclage or oral tocolytics do not reduce the risk of preterm birth in these women^1^, ^102^, ^103^, ^104^, ^105^, ^106^, ^107^, ^108^, ^109^, ^110^, ^111^, ^112^. Early studies of progesterone did not suggest that it was effective in reducing the incidence of preterm birth in twin pregnancy^102^, ^113^, ^114^, ^115^. However, in 2022, an updated IPD meta‐analysis^116^ showed that vaginal progesterone significantly reduced preterm birth < 33 weeks in twin pregnancy with a second‐trimester cervical length ≤ 25 mm (relative risk, 0.60; 95% CI, 0.38–0.95), although the sample size was small (*n* = 95). Composite neonatal morbidity and mortality were also reduced significantly. These findings should be confirmed by an adequately powered RCT.

Although several studies found that cervical cerclage did not reduce the risk of preterm birth in twin pregnancy^105^, ^109^, ^110^, ^112^, ^117^, ^118^, a recent RCT investigating the efficacy of physical‐examination‐indicated cerclage in combination with indomethacin and antibiotics in asymptomatic twin pregnancies with cervical dilation between 1 cm and 4 cm before 24 weeks' gestation^117^, was stopped early due to the significant decrease in preterm birth at all gestational ages, a 50% decrease in preterm birth < 28 weeks and a 78% reduction in perinatal mortality in the cerclage group. A meta‐analysis of RCTs and observational studies suggested that cerclage may also reduce the risk of preterm birth and improve perinatal outcome in asymptomatic women with twin pregnancy and a short cervix (≤ 15 mm) before 24 weeks of gestation^118^ (**EVIDENCE LEVEL: 1+**). An RCT (the PROSPECT study) comparing 200 mg vaginal progesterone or cervical pessary *vs* placebo to prevent early preterm birth in women with a twin pregnancy and cervix < 30 mm is scheduled to finish in 2025.

### Screening, diagnosis and management of FGR in twin pregnancy

#### Diagnostic criteria and investigations for sFGR

- sFGR is conventionally defined as a condition in which one fetus has EFW < 10^th^ centile and the intertwin EFW discordance is ≥ 25% (**GOOD PRACTICE POINT**).
- A discordance cut‐off of 20% seems acceptable to distinguish pregnancies at increased risk of adverse outcome (**GRADE OF RECOMMENDATION: B**).

The definition, assessment and management of FGR is inconsistent among clinicians. If both twins have EFW < 10^th^ centile, the fetuses should be termed SGA. Conventionally, sFGR is a term applied to twin pregnancies in which one fetus has EFW < 10^th^ centile and the intertwin EFW discordance is ≥ 25%^119^, ^120^. The American College of Obstetricians and Gynecologists considers a difference in EFW of 15–25% to constitute discordant fetal growth^120^. A cut‐off of 18% for discordance in birth weight was found to predict adverse outcome optimally^121^. Some clinicians do not take into account the intertwin EFW discordance (and use instead EFW < 10^th^ centile in one twin). Furthermore, the discordance cut‐off most predictive of adverse outcome is likely to vary with gestational age^122^. A discordance cut‐off of 20% seems a pragmatic choice for distinguishing pregnancies at increased risk of adverse outcome. EFW discordance is calculated by the following formula: ((weight of larger twin − weight of smaller twin)/weight of larger twin) × 100 (**EVIDENCE LEVEL: 2**++). According to an expert consensus using the Delphi procedure, EFW < 3^rd^ centile in one twin is sufficient to diagnose sFGR. Additional criteria for the diagnosis of sFGR require at least two out of four parameters in monochorionic twin pregnancies (EFW of one twin < 10^th^ centile, abdominal circumference of one twin < 10^th^ centile, EFW discordance ≥ 25% and UA pulsatility index (PI) of the smaller twin > 95^th^ centile) and at least two out of three parameters in dichorionic twin pregnancies (EFW of one twin < 10^th^ centile, EFW discordance ≥ 25% and UA‐PI of the smaller twin > 95^th^ centile)^123^.

Once a diagnosis has been made, a cause should be sought^120^. This search should include a detailed anomaly scan and screening for viral infections (cytomegalovirus, rubella and toxoplasmosis). Amniocentesis may also be offered to exclude chromosomal abnormalities as a cause of FGR^120^. sFGR in monochorionic twin pregnancy occurs mainly due to unequal sharing of the placental mass and vasculature^124^ (**EVIDENCE LEVEL: 3**).

#### Screening for FGR in twin pregnancy

- A combination of head, abdomen and femur measurements performs best in calculating EFW (**GRADE OF RECOMMENDATION: B**).
- If intertwin discordance is ≥ 25% or the EFW of one twin is < 10^th^ centile, a referral should be made to a specialist fetal medicine center (**GOOD PRACTICE POINT**).

Assessing EFW using ultrasound is less accurate in twin than in singleton pregnancy^125^. EFW charts that include a combination of head, abdomen and femur measurements perform best in both singleton and twin pregnancy^126^. Currently, the charts used to monitor fetal growth in twin pregnancy are the same as those used for singletons. However, there is a reduction in fetal growth in twin compared with singleton pregnancy, particularly in the third trimester^126^. This is particularly marked in MCDA pregnancies. The use of twin‐specific charts is associated with a marked decrease in the diagnosis of SGA or FGR with their associated consequences, without affecting the rate of stillbirth, adverse perinatal outcomes, or long‐term morbidity^127^, ^128^, ^129^, ^130^. A recent study investigated the risk of perinatal mortality, preterm birth, hypertensive disorders of pregnancy and admission to the neonatal unit in twins classified as SGA using twin and/or singleton charts^131^. The study found that twins classified as SGA according to singleton charts but not according to twin charts had similar outcomes to twins classified as appropriate‐for‐gestational age. The authors concluded that the use of singleton charts was associated with misclassification of a large number of twins as being at risk of FGR. Therefore, twin‐specific charts could potentially reduce unnecessary medical interventions prenatally and postnatally. So far, the use of specific twin growth charts has been controversial due to the concern that the reduced growth in the third trimester observed in most twin pregnancies might be caused by some degree of placental insufficiency, warranting close observation (**EVIDENCE LEVEL: 2++**). However, in view of the recent evidence originating from several countries^129^, ^131^, ^132^, the 2022 Canadian guidelines on management of dichorionic twin pregnancies proposed the use of twin charts^133^.

EFW discordance between twins is significantly associated with the risk of perinatal loss^123^, ^130^, ^134^, ^135^. Various thresholds have been used to classify EFW discordance. The Southwest Thames Obstetric Research Collaborative (STORK) found that the 95^th^ centile of EFW discordance was 18.3% at 20 weeks for dichorionic twins, increasing to 21.9% by 30 weeks; for monochorionic twins the equivalent figures were 22.2% at 20 weeks and 25.4% at 30 weeks^136^. A meta‐analysis showed that the risk of stillbirth was increased in dichorionic twins with EFW discordance of ≥ 15% (OR, 9.8; 95% CI, 3.9–29.4) and in monochorionic twins with EFW discordance of ≥ 20% (OR, 2.8; 95% CI, 1.3–5.8), with an increased risk of neonatal death in monochorionic twins with discordance ≥ 25% (OR, 4.66; 95% CI, 1.8–12.4)^130^. Moreover, the optimal threshold for prediction of single IUD changes with increasing gestational age (48% at 28 + 0 to 30 + 6 weeks, 20% at 31 + 0 to 33 + 6 weeks and 14% at 34 + 0 to 36 + 6 weeks)^122^. Therefore, the decision to deliver should also take into account gestational age, chorionicity, Doppler indices and antenatal complications, and not be based on EFW discordance alone. A study evaluating various diagnostic criteria for sFGR identified significant variations in its incidence depending on the criteria applied, highlighting the need for using standardized international diagnostic criteria^137^. Recent updates from the UK National Institute for Health and Care Excellence guidance recommend that EFW discordance should be calculated and documented at every scan from 20 weeks onwards, and UA Doppler performed together with weekly scans if EFW discordance is > 20% or the EFW of one twin is < 10^th^ centile^16^. Further progression to an EFW discordance of ≥ 25% should prompt referral to a specialist fetal medicine unit for assessment, increased fetal surveillance, including fetal Doppler, and planning of delivery when appropriate^1^ (**EVIDENCE LEVEL: 2++**). sFGR in dichorionic twin pregnancy, similar to that in singleton pregnancy, is classified into early (< 32 weeks' gestation) and late (≥ 32 weeks)^138^, while, in monochorionic twin pregnancy, the cut‐off to define early *vs* late sFGR is 24 weeks^137^.

#### Classification of monochorionic twin pregnancy complicated by sFGR

- Classification of sFGR in monochorionic twins has traditionally relied on the pattern of end‐diastolic velocity on UA Doppler (**GOOD PRACTICE POINT**).

**Figure 4:**
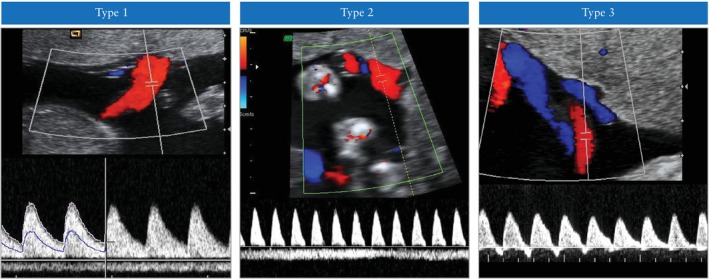
Classification of selective fetal growth restriction in monochorionic twin pregnancy. In Type I, the umbilical artery Doppler waveform has positive end‐diastolic flow, while in Type II there is absent or reversed end‐diastolic flow (AREDF). In Type III, there is a cyclical/intermittent pattern of AREDF.

The Gratacós classification of sFGR in monochorionic twin pregnancy depends on the pattern of end‐diastolic velocity in the UA of the smaller twin (Figure 4)^139^. In Type I, the UA Doppler waveform has positive end‐diastolic flow (EDF). In Type II, there is absent or reversed end‐diastolic flow (AREDF). In Type III, there is a cyclical/intermittent pattern of AREDF. The overall twin survival rate in Type‐I sFGR is greater than 90% (*in‐utero* mortality rates of up to 4%). Type‐II sFGR is associated with a high risk of IUD of the growth‐restricted twin and/or very preterm delivery with associated risk of neurodevelopmental delay if the other twin survives (IUD of either twin in up to 29% of cases and risk of neurological sequelae in up to 15% of cases born prior to 30 weeks). Type‐III sFGR is associated with a 10–20% risk of sudden death of the growth‐restricted fetus, which is unpredictable (even in cases in which ultrasound features have been stable). There is also a high (up to 20%) associated rate of neurological morbidity in the surviving larger twin^119^, ^140^, ^141^, ^142^ (**EVIDENCE LEVEL: 2++**).

#### Managing dichorionic twin pregnancy complicated by sFGR

- In dichorionic twin pregnancies, sFGR should be monitored similarly to growth‐restricted singletons (**GOOD PRACTICE POINT**).

In dichorionic twin pregnancy complicated by sFGR, the timing of delivery should be determined based on a risk–benefit assessment and according to the wishes of the parents, guided by obstetric and neonatal counseling. As these twins have separate circulations, the pregnancy can be followed up similarly to growth‐restricted singleton pregnancy, monitoring for progressive deterioration of UA, MCA and DV Doppler parameters and of biophysical profile scores. In dichorionic twin pregnancy complicated by sFGR, fetal Doppler should be assessed at least every 2 weeks, depending on the severity. These pregnancies should be managed in specialist centers with the relevant expertise. Expectant management until 28–30 weeks can be followed to avoid the risk to the larger twin of iatrogenic prematurity. If death of the smaller twin occurs, there is a risk of preterm birth of 54%, a risk of death of 3% and a risk of neurodevelopmental impairment of 2% for the larger cotwin^143^, ^144^, ^145^. There is limited evidence to guide the gestational age at which delivery is recommended. In the absence of high‐quality twin‐specific evidence, the ISUOG guidelines for the diagnosis and management of FGR in singleton pregnancies^146^ can be followed to inform the decision for delivery based on the condition of the growth‐restricted twin. Accordingly, delivery is indicated between 29 + 0 and 31 + 6 weeks if the DV a‐wave is at or below baseline, or fetal heart rate short‐term variation (STV) is < 3.0 ms; between 32 + 0 and 33 + 6 weeks (permitted after 30 + 0 weeks) if UA‐EDF is reversed or STV is < 3.5 ms; ≥ 34 + 0 weeks (permitted after 32 + 0 weeks) if UA‐EDF is absent or STV is < 4.5 ms; and 36 + 0 weeks onwards if UA‐PI is > 95^th^ percentile or abdominal circumference/EFW is < 3^rd^ percentile^146^.

#### Managing monochorionic twin pregnancy complicated by sFGR

- In monochorionic twin pregnancy complicated by sFGR, fetal Doppler should be assessed at least weekly (**GOOD PRACTICE POINT**).
- If there is a substantial risk of fetal demise of one cotwin before 26 weeks, selective termination may be considered (**GRADE OF RECOMMENDATION: D**).

There is limited evidence to guide the management of monochorionic twin pregnancies affected by sFGR. Options include: conservative management followed by early delivery; laser ablation; or selective termination of the growth‐restricted twin in order to protect the cotwin^147^ (**EVIDENCE LEVEL: 2–**).

In these pregnancies, fetal growth should be assessed at least every 2 weeks, and fetal Doppler (UA and MCA) at least weekly. If the UA Doppler is abnormal, assessment of the DV blood flow should be undertaken. The aim in managing these pregnancies is to prolong the pregnancy at least until viability of the larger twin is achieved, while at the same time avoiding single IUD with its associated potentially serious consequences for the surviving cotwin.

A recent meta‐analysis compared the outcomes following expectant management, fetoscopic laser ablation and selective termination in monochorionic twin pregnancies with sFGR, according to the Gratacós classification^148^. In Type‐I sFGR, 3.1%, 16.7% and 1.0% of cotwins had IUD following expectant management, laser ablation and selective termination, respectively. In Type‐II sFGR, 16.6%, 44.3% and 5.0% of cotwins, respectively, experienced IUD following these treatments, and 89.3%, 100% and 90.6% of surviving fetuses were free of neurological sequelae. In Type‐III sFGR, 13.2%, 32.9% and 0% of cotwins, respectively, experienced IUD after these treatments, and 61.9%, 100% and 98.8% had intact neurological development. The authors concluded that, in severe early‐onset cases, fetal intervention is associated with increased mortality but may reduce perinatal morbidity.

The criteria to define severe sFGR are not clearly established, but early onset and abnormal UA Doppler in the smaller twin, especially if combined with abnormal DV Doppler^149^ and oligohydramnios^150^, have been reported as signs of poor prognosis in observational series^151^.

The Gratacós classification does not take into account the gestational age at diagnosis, variation in UA Doppler in the smaller twin, especially in early gestation, DV Doppler or the coexistence of TTTS or IUD of the smaller twin. In a cohort study of MCDA twin pregnancies followed from the first trimester until birth^137^, in cases of early‐onset sFGR (< 24 weeks' gestation), the incidence of Type‐I, Type‐II and Type‐III sFGR was 81%, 15% and 4%, respectively. In late‐onset (≥ 24 weeks) cases, the corresponding figures were 94%, 6% and 0%, respectively. The incidence of superimposed TTTS was 27% in cases affected by early‐onset sFGR compared with 6% in those with late‐onset sFGR. Therefore, gestational age at diagnosis influences the incidence, type and prognosis of sFGR and should be taken into account. There is debate around whether the Gratacós classification should be modified to include these prognostic factors^152^. Of note, this classification was created for early‐onset and isolated sFGR specifically.

In cases in which Doppler assessment concludes that there is a real risk of fetal demise of one twin before viability (e.g. the smaller twin may weigh < 500 g at 28 weeks), the option of selective termination (or laser ablation, when law does not permit selective termination) should be explored in order to protect the normally grown fetus from serious harm should the smaller twin die *in utero*. Management of these cases is complex and should be coordinated by a tertiary‐level fetal medicine center^147^ (**EVIDENCE LEVEL: 2–**).

The timing of delivery should be decided based on assessment of fetal wellbeing, interval growth, biophysical profile, DV waveform and/or computerized cardiotocography (CTG), when available. However, as the risk of IUD in these pregnancies is increased, delivery might be indicated even before abnormalities in the DV Doppler or computerized CTG become evident. Furthermore, the incidence of severe cerebral injury in monochorionic twin pregnancies complicated by sFGR is approximately 10% and is associated with abnormal UA Doppler, single IUD and low gestational age at birth^141^. Interestingly, the risks of neonatal morbidity (38% *vs* 19%), particularly respiratory distress syndrome (32% *vs* 6%) and cerebral lesions, are higher in the larger than in the smaller twin^153^. A retrospective cohort study assessed, at a median age of 11 years, 44 MCDA pairs that had had sFGR, finding mild impairment in 36% of smaller twins and 11% of larger twins, and severe impairment in 4% of both smaller and larger twins^154^ (**EVIDENCE LEVEL: 2++**).

### Managing the surviving twin after demise of its cotwin

- When single IUD occurs in a twin pregnancy, the woman should be referred to a tertiary‐level center with relevant expertise (**GOOD PRACTICE POINT**).

Following single IUD, the following complications are found in monochorionic and dichorionic pregnancies, respectively^143^, ^144^, ^145^:

– death of the cotwin: 15% and 3%;
– preterm delivery < 34 weeks: 68% and 54%;
– abnormal postnatal cranial imaging of the surviving cotwin: 34% and 16%;
– neurodevelopmental impairment of the surviving cotwin: 26% and 2% (**EVIDENCE LEVEL: 2++**).

The latency from single IUD to preterm birth is inversely proportional to the gestational age at the time of IUD (i.e. shorter interval to birth when single IUD is later in gestation)^144^, ^155^, ^156^. When one monochorionic twin dies *in utero*, the surviving twin may lose part of its circulating volume to the dead twin, leading to potentially severe hypotension in the survivor. This can lead to hypoperfusion of the brain and other organs, which can cause brain damage or death^144^ (**EVIDENCE LEVEL: 2++**).

When single IUD occurs in a monochorionic twin pregnancy, the woman should be managed at a tertiary‐level center with relevant expertise. This should include assessment of fetal Doppler, especially MCA‐PSV, in order to look for signs of fetal anemia in the surviving twin. Conservative management (i.e. continuing the pregnancy) is often the most appropriate course of action. Swift delivery is usually not indicated, because, if the surviving twin suffers any neurological harm, often this has already happened by the time the death has been diagnosed. If the pregnancy is near term, then it makes sense to deliver without delay, but, if it is preterm, prolonging the pregnancy for the benefit of the surviving twin (in terms of increased maturity) is usually recommended. Detailed counseling of the parents is required. This should include an explanation of the risk that there might be significant long‐term morbidity (neurological or otherwise) of the surviving twin but that this damage may have taken place already and urgent delivery may be too late to prevent such harm. In the short term, the surviving twin should be assessed for evidence of ongoing fetal compromise using CTG or MCA Doppler to assess for fetal anemia^157^. If conservative management is chosen, fetal biometry and assessment of UA and MCA Doppler should be scheduled every 2–4 weeks, and delivery should be considered at 34–36 weeks, after a course of maternal steroids. If the MCA‐PSV is normal in the first few days, fetal anemia is unlikely to occur later. Increased MCA‐PSV > 1.5 MoM is associated with, but predicts poorly, cerebral injury after sIUD (sensitivity of 70% for a FPR of 40%)^158^. The fetal brain should be imaged around 4–6 weeks after the death of the cotwin to search for evidence of cerebral morbidity. In cases in which there is strong evidence that the surviving cotwin may have suffered serious neurological harm, late termination of pregnancy should be considered as an option, if the law permits. Neurodevelopmental assessment of the surviving twin at the age of 2 years is recommended. There have been some reports of intrauterine transfusion of an anemic surviving cotwin, but whether this prevents long‐term neurological morbidity is unknown^159^, ^160^, ^161^ (**EVIDENCE LEVEL: 3**).

## COMPLICATIONS UNIQUE TO MONOCHORIONIC TWIN PREGNANCY

Complications which occur only in monochorionic twin pregnancy include TTTS, TAPS, TRAP sequence, monoamniotic pregnancy and conjoined twinning.

### Screening, diagnosis, staging and management of TTTS

Up to one‐third of twin pregnancies are monochorionic. In nearly all monochorionic twins, the placenta contains vascular anastomoses connecting the two fetal circulations. It is the angioarchitecture of these vascular anastomoses that determines the risk profile. Monochorionic twins are at risk of developing TTTS when there is unequal hemodynamic and amniotic fluid balance^162^, ^163^, ^164^, ^165^. The diagnosis of TTTS requires the presence of significant amniotic fluid imbalance. According to the traditional Quintero staging^162^, the ‘donor’ twin has a DVP *≤* 2 cm (oligohydramnios) and the ‘recipient’ twin has a DVP *≥* 8 cm (polyhydramnios). In Europe, the diagnosis of polyhydramnios is made when DVP is ≥ 8 cm at ≤ 20 weeks and ≥ 10 cm after 20 weeks' gestation. A lower cut‐off of DVP of 6 cm has been proposed to diagnose polyhydramnios prior to 16 weeks' gestation^152^. A recent study from the USA questioned the restriction of the definition of TTTS to a DVP for the recipient of ≥ 10 cm beyond 20 weeks as this would potentially exclude 14.5% of patients from laser surgery, the majority of whom had severe TTTS^166^. Size discordance is a common finding but is not essential for the diagnosis. TTTS affects 10–15% of monochorionic twin pregnancies and is associated with increased perinatal mortality and morbidity; if untreated, it leads to fetal demise in up to 90% of cases, with morbidity rates in survivors of over 50%^164^, ^165^. Early diagnosis, however, may allow intervention with fetoscopic laser ablation, which improves the prognosis significantly. Laser treatment in these pregnancies results in 60–70% double survival and 80–90% survival of at least one twin^165^, ^167^, ^168^.

#### Staging of TTTS

- Quintero staging remains the classification system of choice, although it does not always predict accurately outcome or chronological evolution of TTTS (**GOOD PRACTICE POINT**).

TTTS is currently classified using the Quintero staging system (Table 1)^162^, ^163^. However, there is some debate about the validity of Quintero staging of TTTS. It has been noted that Stage‐I disease is not necessarily associated with the best outcomes. For example, some recipient twins in pregnancies categorized as Quintero Stage‐I TTTS may have a degree of cardiac dysfunction^169^, ^170^, ^171^. Another criticism is that it does not represent a chronological order of deterioration; for example, Stage I can become Stage V without passing through Stages II, III and IV, and it does not predict survival well after treatment. While incorporation of additional cardiovascular parameters stratifies additional disease features independent of Quintero staging, these do not improve prediction of outcome following treatment. Nevertheless, Quintero staging remains the most commonly used system for classification of twin pregnancy complicated by TTTS (**EVIDENCE LEVEL: 2+**). Recently, the differentiation between TTTS Stages I *vs* II and III *vs* IV was questioned as it did not have any significant prognostic implication for perinatal survival^172^. Of note, double survival and survival of at least one fetus were significantly lower in cases with Quintero Stages III and IV compared to those with Quintero Stages I and II^172^.

**Table 1:** Quintero staging system for twin‐to‐twin transfusion syndrome^162^

Stage	Classification
I	Polyhydramnios–oligohydramnios sequence: DVP ≥ 8 cm in recipient twin and DVP ≤ 2 cm in donor twin
II	Bladder in donor twin not visible on ultrasound imaging
III	Absent/reversed end‐diastolic flow in the umbilical artery, reversed flow in the ductus venosus or pulsatile flow in the umbilical vein in either twin
IV	Hydrops in one or both twins
V	Death of one or both twins

DVP, deepest vertical pocket.

#### Screening for TTTS

- In monochorionic twin pregnancy, screening for TTTS should start at 16 weeks, with scans repeated every 2 weeks thereafter (**GOOD PRACTICE POINT**).

Monitoring of monochorionic twin pregnancy for the development of TTTS should start with a scan at 16 weeks' gestation, as earlier intervention is not possible; scans should be repeated every 2 weeks thereafter. In a retrospective cohort study of 675 MCDA twins followed from the first trimester, a fortnightly follow‐up scheme detected 90% of TTTS cases in time (i.e. before demise, ruptured membranes or a dilated cervix). The 10% that were detected too late were complicated by fetal demise either prior to 16 weeks or after 26 weeks^173^. A small cohort study of 44 TTTS pregnancies suggested that women who have ultrasound scans less often than fortnightly may be more likely to have advanced stages of TTTS upon diagnosis^174^. However, this was not confirmed in a larger cohort study of 82 TTTS pregnancies, in which the interval between the last scan and TTTS diagnosis did not differ between those diagnosed with TTTS Stages I–II and those with Stages III–IV. However, advanced stages presented earlier and, in the majority of cases, abnormal Doppler findings preceded the TTTS diagnosis, suggesting that more frequent follow‐up may not result in an earlier stage of disease at diagnosis. As discussed above, Quintero staging did not reflect progressive worsening of the disease^173^ (**EVIDENCE LEVEL: 2+**). Several studies have attempted to identify first‐trimester markers of later complications such as TTTS in monochorionic pregnancy, but a recent meta‐analysis found that, as yet, this is not possible^66^. For monochorionic twin pregnancy, at every scan, the operator should note and record evidence of membrane folding and measure the DVP of amniotic fluid for each fetus. If there is significant inequality in DVP or there is membrane infolding, then more frequent ultrasound surveillance may be warranted. TTTS is far less common in MCMA, compared with MCDA, twin pregnancy; the ultrasound diagnostic features in MCMA pregnancies include polyhydramnios in the common amniotic sac and discordant bladder sizes.

#### Prognosis for monochorionic twin pregnancy with amniotic fluid discordance

- Monochorionic twin pregnancies with uncomplicated amniotic fluid discordance can be followed up on a weekly basis to exclude progression to TTTS (**GOOD PRACTICE POINT**).

Monochorionic twin pregnancies with amniotic fluid discordance between the twins (defined as a difference of 4 cm or more in their DVPs) which does not fulfil the DVP ≥ 8 cm/≤ 2 cm criterion (in other words, DVP falls within the ‘normal’ range), and which have normal UA Doppler measurements, are associated with a good outcome (93% overall survival) and a low risk (14%) of progression to severe TTTS^175^, ^176^, ^177^. However, it is common practice for these pregnancies to be followed up on a weekly basis initially, to ensure that there is no progression to TTTS (**EVIDENCE LEVEL: 2+**).

#### Treatment of TTTS

- Laser ablation is the treatment of choice for TTTS at Quintero Stages II, III and IV (**GRADE OF RECOMMENDATION: A**).
- Conservative management with close surveillance may be considered for asymptomatic women with Quintero Stage I and a long cervix (> 15 mm) (**GRADE OF RECOMMENDATION: A**).
- When laser treatment is not available, serial amnioreduction is an acceptable alternative after 26 weeks' gestation (**GRADE OF RECOMMENDATION: A**).

TTTS diagnosed before 26 weeks of gestation is best treated by laser ablation, as the evidence suggests that this leads to better outcomes compared with amnioreduction or septostomy^165^, ^177^ (**EVIDENCE LEVEL: 1+**). It is generally accepted that Quintero Stages II and above will require treatment. If laser ablation expertise is not available, amnioreduction is an acceptable alternative in pregnancies diagnosed after 26 weeks of gestation^165^. There is, however, evidence that laser ablation is the best form of treatment for TTTS, regardless of whether it is diagnosed early (before 16 weeks) or late (after 26 weeks' gestation)^177^, ^178^.

Management of Quintero Stage‐I TTTS has been controversial. A meta‐analysis of Stage‐I TTTS showed a similar rate of survival of at least one twin with expectant management (87%; 95% CI, 69–98%), amnioreduction (86%; 95% CI, 76–94%) or laser photocoagulation (81%; 95% CI, 69–90%), with a progression rate of 27% (95% CI, 16–39%)^179^. The North American Fetal Therapy Network found that both amnioreduction (OR, 0.11; 95% CI, 0.02–0.68) and laser photocoagulation (OR, 0.07; 95% CI, 0.01–0.37) reduced the risk of no survivors, and was protective against poor outcome (OR, 0.12; 95% CI, 0.03–0.44)^180^ (**EVIDENCE LEVEL: 2+**). A recent multicenter RCT^181^ randomized asymptomatic women with Stage‐I TTTS at 16–26 weeks' gestation and a long cervix (> 15 mm) to either laser surgery or expectant management. There was no difference between the two groups in survival at 6 months without severe neurological morbidity (**EVIDENCE LEVEL: 1+**). However, 60% of conservatively managed twins eventually required laser surgery and these cases had a non‐significant trend for lower intact survival. If conservative management is chosen for Quintero Stage‐I TTTS, worsening polyhydramnios, maternal discomfort and shortening of the cervical length are considered ‘rescue’ criteria signaling a need to proceed with fetoscopic laser ablation, and whether there is access to a laser center should be taken into consideration. In a systematic review of the management of pregnancies with Stage‐I TTTS, overall survival appeared to be similar for those undergoing laser therapy or conservative management (85% and 86%, respectively), but was somewhat lower for those undergoing amnioreduction (77%)^182^ (**EVIDENCE LEVEL: 2–**).

Following laser treatment, the recurrence rate of TTTS is up to 14%, which is likely to be due to anastomoses missed at the time of the initial laser treatment^183^ (**EVIDENCE LEVEL: 2**–). The risk of recurrence of TTTS and occurrence of TAPS is reduced by use of the Solomon technique (equatorial laser dichorionization) compared with the highly‐selective technique^167^, ^168^ (**EVIDENCE LEVEL: 1+**). In a recent meta‐analysis, the Solomon technique had a significantly higher survival rate and lower recurrence rate of TTTS, but was associated with an increased risk of placental abruption and earlier gestational age at delivery^184^.

Another option for the management of severe TTTS is selective termination of pregnancy using bipolar diathermy, intrafetal laser ablation or RFA of one of the umbilical cords. This means that the most affected fetus is sacrificed in the hope of protecting the other twin from death or cerebral damage. Rarely, parents may opt for termination of the entire pregnancy.

#### Follow‐up and optimal gestational age for delivery in twin pregnancy with TTTS

- A common practice is weekly ultrasound assessment after treatment of TTTS, reducing to alternate weeks following clinical evidence of resolution (**GOOD PRACTICE POINT**).
- In case of demise of one fetus (post‐laser), brain imaging of the surviving cotwin should be considered 4–6 weeks later, and neurodevelopmental assessment should take place at 2 years of age (**GOOD PRACTICE POINT**).

There is no evidence to guide frequency of ultrasound follow‐up after treatment of TTTS. However, treatment should result in normalization of amniotic fluid by 14 days^185^. Cardiac dysfunction generally normalizes in the recipient within 1 month, while the donor suffers a temporary impairment of cardiac function^186^ (**EVIDENCE LEVEL: 2+**). A common practice is weekly ultrasound assessment for the first 2 weeks after treatment, reducing to alternate weeks following clinical evidence of resolution. Each ultrasound scan should assess the DVP, biometry (every 2 weeks), and UA, MCA (PSV) and DV Doppler in both fetuses. Right outflow stenotic lesions are common in these twins, more commonly in the recipients^187^, while 8% of all twins will have pulmonary artery stenosis at the age of 10 years^188^ and more than 8% suffer antenatal brain damage^189^ (**EVIDENCE LEVEL: 2–**). There should be a detailed assessment of the brain, heart and limbs (due to risk of amputation secondary to thrombi or amniotic bands) during these follow‐up scans. Functional heart problems and antenatal cerebral lesions may become obvious only in the third trimester. Some fetal medicine centers offer fetal brain MRI at 30 weeks to all survivors after laser treatment, in order to detect brain anomalies such as migration and proliferation disorders. However, evidence to support this practice is limited and the specificity of diagnosis and how this translates into long‐term neurological morbidity is unknown^190^. A recent meta‐analysis found that the overall incidence of antenatally diagnosed fetal brain abnormality in twin fetuses complicated by TTTS treated with laser surgery is around 2%, and that it is mainly ischemic in nature in approximately one‐third of cases^191^.

There is limited evidence on the optimal timing and route of delivery for monochorionic twins previously treated for TTTS, but the general consensus is that this should be at 34 weeks of gestation, after a course of steroids^192^. However, it is also reasonable to adopt a similar strategy as that for all monochorionic twins, with delivery at 34 weeks of gestation for persisting abnormalities and up to 37 weeks where there is complete resolution. The optimal route of delivery following laser therapy has not been determined. Twin pregnancies treated by laser for TTTS should be considered as high risk for adverse outcomes, even if normalization of the amniotic fluid occurs (**EVIDENCE LEVEL: 2–**). In pregnancies complicated by demise of one fetus (post‐laser), brain imaging should be considered 4–6 weeks later, and neurodevelopmental assessment should take place at the age of 2–3 years.

#### Risk of brain abnormalities and neurodevelopmental delay in twin pregnancy with TTTS

Monochorionic twin pregnancies complicated by TTTS, single IUD, sFGR or TAPS are at increased risk of brain abnormalities and neurodevelopmental disability^141^, ^144^, ^193^, ^194^. In pregnancies complicated by TTTS, cerebral abnormalities were reported in 5% of those undergoing laser photocoagulation, 14% following serial amnioreduction and 21% following expectant management^194^ (**EVIDENCE LEVEL: 2–**). Both donors and recipients are at risk of developing either ischemic or hemorrhagic lesions^194^. At a median age of 34 months following laser treatment for TTTS, 7% of the children had major neurological abnormalities^195^, ^196^ (**EVIDENCE LEVEL: 2–**). The neurodevelopmental outcome at 6 years of age was similar to that at 2 years and 10 months, with 9% of the children experiencing major neurodevelopmental delay^197^ (**EVIDENCE LEVEL: 2–**). The risk of long‐term neurodevelopmental impairment likely decreases with increased clinical experience^198^, ^199^.

### Screening, diagnosis and management of TAPS

- The prenatal diagnosis of TAPS is based on the finding of discordant MCA Doppler abnormalities (**GRADE OF RECOMMENDATION: D**).
- There is limited evidence from observational studies regarding the outcome and optimal management of TAPS; therefore, treatment options should be individualized and discussed with parents (**GOOD PRACTICE POINT**).

TAPS is a complication of monochorionic twin pregnancy that occurs when there is significant intertwin discordance in hemoglobin levels and reticulocyte counts in the absence of significant disparity in amniotic fluid volume. Understanding of the natural history and fetal and neonatal implications of TAPS in monochorionic pregnancy is still evolving. Moreover, the optimal treatment and frequency and mode of surveillance have yet to be established. The incidence of TAPS occurring spontaneously in MCDA twins is up to 5%. However, it may complicate up to 13% of cases with TTTS following laser ablation^183^. TAPS is believed to be due to the presence of miniscule (< 1 mm) arteriovenous anastomoses which allow slow transfusion of blood from the donor to the recipient, leading to highly discordant hemoglobin concentrations at birth (**EVIDENCE LEVEL: 3**). Postnatally, TAPS is diagnosed based on the finding of chronic anemia (including reticulocytosis) in the donor and polycythemia in the recipient. The criteria for postnatal diagnosis include a difference in hemoglobin concentration between the twins of > 8 g/dL and at least one of either reticulocyte count ratio > 1.7 or small vascular anastomoses (< 1 mm in diameter) in the placenta^200^, ^201^. Prenatally, TAPS is diagnosed based on the finding of discordant MCA Doppler abnormalities, including MCA‐PSV > 1.5 MoM in the donor, suggesting fetal anemia, and MCA‐PSV < 1.0 MoM in the recipient, suggesting polycythemia. These diagnostic criteria have a sensitivity of 46% and specificity of 100% for postnatal TAPS, with positive and negative predictive values of 100% and 70%, respectively^202^. Recent studies found that recipient twins with MCA‐PSV > 1.0 MoM could still be polycythemic at birth; therefore, various alternative diagnostic criteria have been proposed^203^, ^204^. As a result, a Delphi consensus group was convened to establish unified criteria^205^. The expert panel agreed that cut‐offs of MCA‐PSV ≥ 1.5 MoM in the donor twin and ≤ 0.8 MoM in the recipient twin, or a delta MCA‐PSV between the twins of ≥ 1.0 MoM, should be used to achieve an antenatal diagnosis of TAPS. However, the diagnostic criteria with the optimal DR and outcome, and the fewest unnecessary interventions, have yet to be established.

Additional ultrasound findings are observed in over 80% of TAPS pregnancies and include differences in placental echogenicity and thickness, with a bright, thickened section associated with the donor and an echolucent, thin section associated with the recipient. The polycythemic twin might have a ‘starry‐sky’ appearance of the liver pattern due to diminished echogenicity of the liver parenchyma and increased brightness of the portal venule walls. The antenatal and postnatal severity‐based staging classifications are shown in Table 2, ^200^, ^201^ (**EVIDENCE LEVEL: 3**).

**Table 2:** Antenatal and postnatal staging of twin anemia–polycythemia sequence (TAPS)^200^, ^201^

Stage	Antenatal staging	Postnatal staging: intertwin Hb diff (g/dL)
1	Donor MCA‐PSV > 1.5 MoM and recipient MCA‐PSV < 1.0 MoM, without other signs of fetal compromise	> 8.0
2	Donor MCA‐PSV > 1.7 MoM and recipient MCA‐PSV < 0.8 MoM, without other signs of fetal compromise	> 11.0
3	Stage 1 or 2 and cardiac compromise in donor (UA‐AREDF, UV pulsatile flow, or DV increased or reversed flow)	> 14.0
4	Hydrops of donor	> 17.0
5	Death of one or both fetuses preceded by TAPS	> 20.0

AREDF, absent or reversed end‐diastolic flow; diff, difference; DV, ductus venosus; Hb, hemoglobin; MCA, middle cerebral artery; MoM, multiples of median; PSV, peak systolic velocity; UA, umbilical artery; UV, umbilical vein.

The outcome of twin pregnancies complicated by TAPS is variable. Severe TAPS may result in the IUD of both twins. At the other end of the spectrum, mild TAPS may still allow the birth of two healthy neonates (apart from a significant difference in hemoglobin level between the two)^206^. It appears that the main neonatal morbidity is anemia (requiring transfusion) and polycythemia (possibly requiring partial exchange transfusion)^207^. However, cases of severe cerebral damage have been reported in TAPS neonates^208^. Evidence suggests that, in monochorionic twins complicated by TAPS, the risk of neurodevelopmental delay is increased^209^. Long‐term neurodevelopmental follow‐up has found neurodevelopmental impairment in 9% and mild–moderate cognitive delay in 17% babies that developed TAPS after laser for TTTS^209^, and in 26% of survivors of spontaneous TAPS^210^. The risk of impairment is higher in donors than in recipients. Also, after spontaneous TAPS, bilateral deafness was identified in 15% of donors and in none of the recipients^210^. Therefore, brain imaging during the third trimester, neonatal auditory screening and neurodevelopmental assessment at the age of 2 years are recommended (**EVIDENCE LEVEL: 2+**).

The perinatal outcomes of twin pregnancies complicated by TAPS, according to whether it was spontaneous or post‐laser for TTTS, have been reported, according to the management options, by the TAPS multicenter registry^206^, ^211^, ^212^. In a meta‐analysis focusing on the outcomes of these pregnancies, post‐laser TAPS was associated with worse perinatal outcome compared with spontaneous TAPS^213^. The management options depend on the gestational age at diagnosis, parental choice, severity of the disease and technical feasibility of intrauterine therapy. Therefore, the management of twin pregnancies complicated by TAPS should be individualized. The most common options include: conservative management, early delivery, laser ablation, intrauterine blood transfusion for the anemic twin, or combined intrauterine blood transfusion for the anemic twin and partial exchange transfusion to dilute the blood of the polycythemic twin^214^. In order to screen for TAPS, MCA‐PSV should be measured in all monochorionic twin pregnancies from 20 weeks onwards in both fetuses, and during the follow‐up of cases treated for TTTS. Prevention of TAPS by modification of the fetoscopic laser ablation technique remains the best way to prevent morbidity^168^, ^215^ (**EVIDENCE LEVEL: 2++**).

### Management of TRAP sequence

- The chances of survival of the pump twin may be increased by the use of minimally invasive techniques (e.g. cord coagulation, cord ligation and photocoagulation of the anastomoses, as well as intrafetal methods) (**GRADE OF RECOMMENDATION: D**).

**Figure 5:**
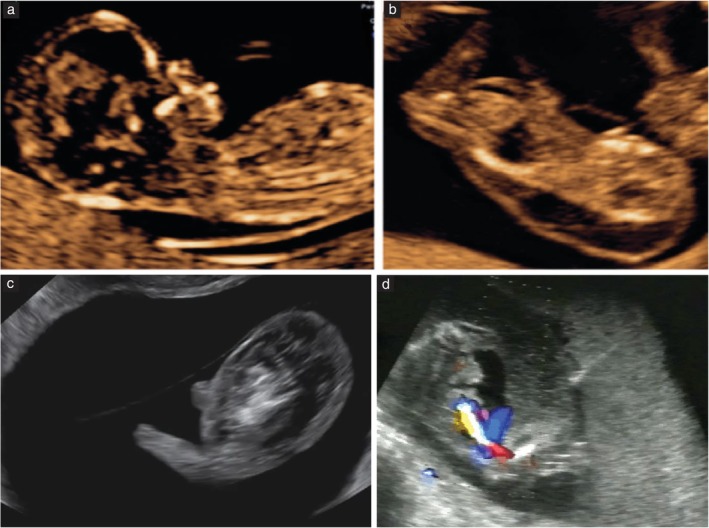
(a) Midsagittal ultrasound image of pump twin in a pregnancy affected by twin reversed arterial perfusion (TRAP) sequence. (b,c) Sagittal views of TRAP mass. (d) Intrafetal laser treatment as a means to arrest the flow in the TRAP mass. The needle is positioned, under ultrasound guidance, in the TRAP mass in the fetal pelvis near the cord insertion.

TRAP sequence is a rare complication of monochorionic twin pregnancy (2.5% of monochorionic twin pregnancies and 1 in 15 000 pregnancies overall)^216^. It is characterized by the presence of a TRAP or acardiac mass perfused by an apparently normal (pump) twin^217^ (Figure 5). The perfusion occurs in a retrograde fashion through arterioarterial anastomoses, usually through a common cord insertion site^218^. This characteristic vascular arrangement predisposes to a hyperdynamic circulation and progressive high‐output cardiac failure in the pump twin^218^. The risk of demise of the pump fetus in TRAP sequence managed conservatively is up to 30% by 18 weeks' gestation^219^ (**EVIDENCE LEVEL: 3**).

Different minimally invasive techniques, such as cord coagulation, cord ligation and photocoagulation of the anastomoses, as well as intrafetal methods, such as RFA and intrafetal laser ablation, are performed as a means of preventing the demise of the pump twin (Figure 5)^217^. The survival rate of the pump twin using these treatment modalities is approximately 80%. TRAP sequence pregnancies may be treated prophylactically by an invasive procedure or they may be monitored serially, with the aim of undertaking intrauterine therapy only if cardiac strain becomes evident in the pump twin or there is increased perfusion (including the occurrence of polyhydramnios) and growth of the TRAP mass (the size can be assessed using estimated weight formulae or as a ratio of the size of the acardiac twin to that of the pump twin)^217^.

Therefore, careful monitoring and ultrasound follow‐up in a specialist fetal medicine center is indicated. However, close monitoring with ultrasound and Doppler does not prevent sudden demise. When treatment is necessary, there may be benefit of intervention before 16 weeks' gestation^220^. The rate of preterm birth before 32 weeks' gestation is approximately 10%^220^. The gestational age at treatment relates inversely to the gestational age at birth. Therefore, survival might be improved by elective intervention at 12–14 weeks' gestation^221^. However, it is important to acknowledge the observational nature of this evidence and the small size of the case series, which does not allow for the assessment of fetal loss rates compared with those following later intervention (**EVIDENCE LEVEL: 3**). This uncertainty has led to the development of the TRAP Intervention Study (TRAPIST), a multicenter RCT comparing early (12–14 weeks) *vs* late (16–18 weeks) intervention for TRAP sequence, which is currently ongoing (https://clinicaltrials.gov/ct2/show/NCT02621645). There is currently no consensus on the timing of birth in TRAP sequence following expectant or active management; therefore, an individualized approach should be adopted, based on the success of treatment, fetal Doppler findings and cardiac stability of the pump twin.

### Management of MCMA twins

- Umbilical cord entanglement is almost always present in MCMA twins (**GRADE OF RECOMMENDATION: D**).
- Delivery by Cesarean section is recommended at 32–34 gestational weeks (**GRADE OF RECOMMENDATION: D**).

MCMA twin pregnancies constitute approximately 5% of monochorionic twin pregnancies^222^. The reported perinatal loss rate before 16 weeks' gestation is as high as 50%^223^ (**EVIDENCE LEVEL: 3**). Most losses are attributable to fetal abnormalities and spontaneous miscarriage^223^ (**EVIDENCE LEVEL: 3**). The management of these pregnancies may be complex and should take place in centers with the relevant expertise. The overall loss rate has improved from 40% in the older literature^224^, ^225^, ^226^ to 10–15% in more recent studies^227^ (**EVIDENCE LEVEL: 2–**). In a cohort study including 98 MCMA twin pregnancies, the perinatal mortality rate (from 20 weeks of gestation until 28 days of age) was 19%^228^. However, the rate was 17% after exclusion of fetuses with a lethal anomaly. After 32 weeks of gestation, only two (4%) pregnancies were complicated by perinatal mortality. The incidence of TTTS and cerebral injury was 6% and 5%, respectively^228^ (**EVIDENCE LEVEL: 2+**). Evidence suggests that MCMA twin pregnancies are at increased risk of IUD compared with other types of twin pregnancy and should be delivered by Cesarean section between 32 and 34 weeks of gestation (**EVIDENCE LEVEL: 3**)^16^. This is based on the finding that, after 32 + 4 weeks' gestation, the risk of IUD is greater in ongoing MCMA pregnancy compared with the risk of non‐respiratory neonatal complications when the twins are delivered^229^. Individualized assessment of these pregnancies should inform the timing of delivery.

A recent meta‐analysis showed that inpatient monitoring was associated with a 3% risk of IUD (95% CI, 1.4–5.2%), while outpatient management had a higher IUD risk of 7.4% (95% CI, 4.4–11.1%)^230^. However, a multicenter cohort study^231^ found no significant difference in perinatal mortality between inpatient and outpatient management groups of MCMA twins (adjusted OR, 0.21; 95% CI, 0.04–1.17) (**EVIDENCE LEVEL: 2+**). This question, therefore, remains unresolved.

It is important to realize that umbilical cord entanglement is present in almost all monoamniotic twins evaluated systematically by ultrasound and color Doppler^232^. A systematic review including a total of 114 monoamniotic twin sets (228 fetuses) with cord entanglement concluded that cord entanglement alone does not contribute to perinatal morbidity and mortality in monoamniotic twin pregnancies^227^. Moreover, the presence of an UA notch, without other signs of fetal deterioration, is not indicative of adverse perinatal outcome^233^ (**EVIDENCE LEVEL: 2 –**).

In MCMA twin pregnancies undergoing selective reduction (because of discordant anomaly, TRAP sequence, severe TTTS or sFGR), cord occlusion and transection are recommended to prevent fetal demise of the other twin due to cord accidents^234^, ^235^, ^236^, ^237^. The perinatal outcomes are similar to those of discordant MCDA twins treated with cord occlusion. However, the rate of preterm prelabor rupture of the membranes is higher and gestational age at delivery is lower in MCMA compared with MCDA pregnancy (**EVIDENCE LEVEL: 3**).

### Diagnosis and management of conjoined twins

Conjoined twins are very rare, occurring in approximately 1 in 100 000 pregnancies (1% of monochorionic twin pregnancies). Conjoined twins are always MCMA twin pregnancies. Diagnosis with ultrasound in the first trimester is now the norm (on visualizing close and fixed apposition of the fetal bodies, with fusion of the skin lines at some point). A series of 14 cases from a single referral center reported that, following diagnosis, 20% of parents opted for termination and 10% of fetuses died *in utero* ^238^. Among those opting to continue the pregnancy, survival to discharge was only around 25%, and the majority of these had significant morbidity.

The classification of conjoined twins depends on the site of the union. The most common form is thoracopagus, in which the twins face each other and have junctions between the chest and abdomen, often with conjoined livers, hearts and intestinal structures^238^.

In ongoing pregnancies, detailed expert ultrasound imaging (with or without MRI) is important in order to detail the cardiovascular (and other) anatomy of the twins as far as possible prior to delivery. Although vaginal delivery of conjoined twins has been reported, there is a significant risk of obstructed labor, dystocia and uterine rupture, so delivery by elective Cesarean section is now the rule^239^. Such pregnancies should be assessed at a fetal medicine referral center, with multidisciplinary assessment and counseling. The pregnancy must be delivered at a center with expertise in the postnatal medical and surgical management of such cases, with the option for neonatal palliative care. There are associated high rates of postnatal mortality and there is almost always morbidity.

## GUIDELINE AUTHORS

**A. Khalil**, Fetal Medicine Unit, St George's Hospital, St George's University of London, London, UK.

**A. Sotiriadis**, Second Department of Obstetrics and Gynaecology, Aristotle University of Thessaloniki, Thessaloniki, Greece.

**A. Baschat**, The Johns Hopkins Center for Fetal Therapy, Baltimore, MD, USA.

**A. Bhide**, Fetal Medicine Unit, St George's Hospital, St George's University of London, London, UK.

**E. Gratacós**, BCNatal, Hospital Clinic and Hospital Sant Joan de Deu, University of Barcelona, IDIBAPS and CIBERER, Barcelona, Spain.

**K. Hecher**, Department of Obstetrics and Fetal Medi‐ cine, University Medical Center Hamburg‐Eppendorf, Hamburg, Germany.

**L. Lewi**, Department of Obstetrics and Gynecology, Uni‐ versity Hospitals Leuven, Leuven, Belgium.

**L. J. Salomon**, Hopital Necker‐Enfants Malades, AP‐HP, Université Paris Descartes, Paris, France.

**B. Thilaganathan**, Fetal Medicine Unit, St George's Hos‐ pital, St George's University of London, London, UK.

**Y. Ville**, Hospital Necker‐Enfants Malades, AP‐HP, Uni‐ versité Paris Descartes, Paris, France.

## CITATION

These Guidelines should be cited as: ‘Khalil A, Sotiriadis A, Baschat A, Bhide A, Gratacós E, Hecher K, Lewi L, Salomon LJ, Thilaganathan B, Ville Y. ISUOG Practice Guidelines (updated): role of ultrasound in twin pregnancy. *Ultrasound Obstet Gynecol* 2025;65(2):253‐276.

## Data Availability

Data sharing not applicable to this article as no datasets were generated or analysed during the current study.

## APPENDIX 1 Grades of recommendation and levels of evidence used in ISUOG guidelines

**Table:**

Classification of evidence levels	
*1*++	*High‐quality meta‐analyses, systematic reviews of randomized controlled trials or randomized controlled trials with very*
	low risk of bias
1+	Well‐conducted meta‐analyses, systematic reviews of randomized controlled trials or randomized controlled trials with
	low risk of bias
1–	Meta‐analyses, systematic reviews of randomized controlled trials or randomized controlled trials with high risk of bias
2++	High‐quality systematic reviews of case–control or cohort studies or high‐quality case–control or cohort studies with
	very low risk of confounding, bias or chance and high probability that the relationship is causal
2+	Well‐conducted case–control or cohort studies with low risk of confounding, bias or chance and moderate probability
	that the relationship is causal
2–	Case–control or cohort studies with high risk of confounding, bias or chance and significant risk that the relationship is
	not causal
3	Non‐analytical studies, e.g. case reports, case series
4	Expert opinion
*Grades of recommendation*	
A	At least one meta‐analysis, systematic review or randomized controlled trial rated as 1++ and applicable directly to the target population; or systematic review of randomized controlled trials or a body of evidence consisting principally of studies rated as 1+ applicable directly to the target population and demonstrating overall consistency of results
B	Body of evidence including studies rated as 2++ applicable directly to the target population and demonstrating overall consistency of results; or extrapolated evidence from studies rated as 1++ or 1+
C	Body of evidence including studies rated as 2+ applicable directly to the target population and demonstrating overall consistency of results; or extrapolated evidence from studies rated as 2++
D	Evidence of level 3 or 4; or evidence extrapolated from studies rated as 2+
Good practice point	Recommended best practice based on the clinical experience of the guideline development group
